# Beyond the CMSSM without an accelerator: proton decay and direct dark matter detection

**DOI:** 10.1140/epjc/s10052-015-3842-6

**Published:** 2016-01-05

**Authors:** John Ellis, Jason L. Evans, Feng Luo, Natsumi Nagata, Keith A. Olive, Pearl Sandick

**Affiliations:** Theoretical Physics and Cosmology Group, Department of Physics, King’s College London, Strand, London, WC2R 2LS UK; TH Division, Physics Department, CERN, 1211 Geneva 23, Switzerland; William I. Fine Theoretical Physics Institute, School of Physics and Astronomy, University of Minnesota, Minneapolis, MN 55455 USA; Kavli IPMU (WPI), UTIAS, University of Tokyo, Kashiwa, Chiba 277-8583 Japan; Department of Physics and Astronomy, University of Utah, Salt Lake City, UT 84112 USA

## Abstract

We consider two potential non-accelerator signatures of generalizations of the well-studied constrained minimal supersymmetric standard model (CMSSM). In one generalization, the universality constraints on soft supersymmetry-breaking parameters are applied at some input scale $M_{\mathrm{in}}$*below* the grand unification (GUT) scale $M_{\mathrm{GUT}}$, a scenario referred to as ‘sub-GUT’. The other generalization we consider is to retain GUT-scale universality for the squark and slepton masses, but to relax universality for the soft supersymmetry-breaking contributions to the masses of the Higgs doublets. As with other CMSSM-like models, the measured Higgs mass requires supersymmetric particle masses near or beyond the TeV scale. Because of these rather heavy sparticle masses, the embedding of these CMSSM-like models in a minimal SU(5) model of grand unification can yield a proton lifetime consistent with current experimental limits, and may be accessible in existing and future proton decay experiments. Another possible signature of these CMSSM-like models is direct detection of supersymmetric dark matter. The direct dark matter scattering rate is typically below the reach of the LUX-ZEPLIN (LZ) experiment if $M_{\mathrm{in}}$ is close to $M_{\mathrm{GUT}}$, but it may lie within its reach if $M_{\mathrm{in}} \lesssim 10^{11}$ GeV. Likewise, generalizing the CMSSM to allow non-universal supersymmetry-breaking contributions to the Higgs offers extensive possibilities for models within reach of the LZ experiment that have long proton lifetimes.

## Introduction

Supersymmetry remains a favored extension of the Standard Model (SM), despite its non-appearance during Run 1 of the LHC [1–5]. Indeed, the discovery of a 125-GeV Higgs boson at the LHC [6, 7] has supplemented the traditional arguments for supersymmetry, including the naturalness of the electroweak scale, the unification of the fundamental interactions and the existence of a cold dark matter candidate (if *R*-parity is conserved). The minimal supersymmetric extension of the SM (MSSM) predicts the existence of a Higgs boson with mass $m_{ h} \lesssim 130$ GeV, and is a prime example of new physics capable of stabilizing the electroweak vacuum for $m_{ h} \sim 125$ GeV [8]. Moreover, global fits in the framework of simple supersymmetric models suggest that the couplings of the lightest supersymmetric Higgs boson should be very similar to those of the Higgs boson in the SM, as is indicated by the ATLAS and CMS experiments [9–12]. When the supersymmetric particle masses are large, which is the case we consider, the Higgs couplings become even more like the SM couplings.

If these arguments are valid, the following questions must be answered: which supersymmetric model is found in *Nature*, and how may it be tested? To begin to answer these questions, we focus here on the MSSM, and more specifically on constrained versions in which the soft supersymmetry-breaking scalar masses $m_0$, gaugino masses $m_{1/2}$ and trilinear terms $A_0$ are assumed to have universal values at some high input mass scale $M_{\mathrm{in}}$. Typically, $M_{\mathrm{in}}$ is chosen to be at the grand-unified-theory (GUT) scale, a scenario called the constrained MSSM (CMSSM) [13–38], in which the ratio of Higgs vacuum expectation values, $\tan \beta$, is a free parameter.

In order to find models with less-constrained dark matter scenarios and simultaneously a sufficiently long lifetime for the proton, we focus here on two one-parameter extensions of the CMSSM: ‘sub-GUT’ models [39–41] in which $M_{\mathrm{in}} < M_{\mathrm{GUT}}$ is free, and the NUHM1 [42–44], in which the two Higgs soft masses are equal at the input scale, $m_1=m_2$, but are allowed to differ from $m_0$. We will also discuss ‘sub-GUT’ models obtained from minimal supergravity (mSUGRA), which are more constrained than the CMSSM, since the gravitino mass $m_{3/2} = m_0$ and the trilinear and bilinear soft supersymmetry-breaking terms are related: $A_0 - B_0 = m_0$ [45–47]. Since mSUGRA models have one fewer parameter, $\tan \beta$ is no longer free. Sub-GUT mSUGRA models have the same number of free parameters as in the CMSSM, but viable models can readily be found.

Although the standard CMSSM with $M_{\mathrm{in}}=M_{\mathrm{GUT}}$ is still viable, there remain only restricted regions of the parameter space of the CMSSM (and, a fortiori, of the more restrictive mSUGRA model) in which a successful prediction for $m_{ h}$ can be reconciled with the measured cold dark matter density [37, 38, 48–80]. The parameter spaces of these models become more restricted when they are embedded in an SU(5) GUT, because they tend to have a proton lifetime which is shorter than the current experimental limits [81–83], even if the supersymmetric sparticle masses are rather heavy.

These problems can be avoided, however, if $M_{\mathrm{in}}$ is identified with some scale lower than the typical GUT scale. An effective scale of supersymmetry breaking significantly below the GUT scale, $M_{\mathrm{in}}<M_{\mathrm{GUT}}$, is not without theoretical motivation. For example, mirage unification models [84–94] and other scenarios such as [95] give exactly such boundary conditions for the soft supersymmetry-breaking parameters. Phenomenologically, these sub-GUT models with $M_{\mathrm{in}}<M_{\mathrm{GUT}}$ have been shown to have an appropriate cold dark matter density in a considerably larger parameter space [51]. As could be expected, a significant part of this parameter space contains points that are compatible with the LHC measurement of $m_{ h}$ and other phenomenological constraints, such as the non-detection of supersymmetric particles at the LHC [1–5].^1^ This reduced tension in sub-GUT models is due to the reduced running of the soft masses, which leads to a sparticle spectrum that is, in general, more compressed than models with $M_{\mathrm{in}} = M_{\mathrm{GUT}}$. Moreover, this compression of the spectrum leads to more avenues for coannihilation [98], which is effective in reducing the relic neutralino density into the range allowed by cosmology.

Similarly, if the Higgs soft masses are allowed to differ from the soft masses of the matter scalars, as in the NUHM, there are more viable options for dark matter. In both the CMSSM and the NUHM, the Higgs mixing mass, $\mu$, and the pseudoscalar mass, $m_A$, are determined through the minimization of the Higgs potential. However, either $\mu$ and/or $m_A$ can be traded for the Higgs soft mass, which can be calculated using the minimization of the Higgs potential.

Here we examine two potential non-accelerator observables in the contexts of these less-constrained models: the proton lifetime and the elastic scattering cross section for the direct detection of dark matter.

In [99], the proton lifetime was computed by renormalization group (RG) running the gauge couplings up to the GUT scale, defined to be where the two electroweak couplings are equal. The imperfection in the unification of the electroweak couplings with the strong coupling was then used to determine the size of the color-triplet Higgs threshold, which then determined the color-triplet Higgs mass [100–102]. Using this procedure with sub-TeV stops and Higgsinos and decoupled first- and second-generation sfermions, it was shown that the lifetime of the proton was shorter than the experimental constraints. Since the experimental constraints are now stronger, this problem has become even worse.

This problem can be avoided in many ways. One particularly simple way is to include an additional pair of $\mathbf{5}$ and $\bar{\mathbf{5}}$ Higgs boson supersymmetric multiplets that do not couple to any of the SM fields. Below the SU(5)-breaking scale, the colored and flavored Higgs mass become free parameters. If the portion of the Higgs supersymmetric multiplet that has SU(3) charges is lighter than the portion with SU(2) charges, the thresholds in the couplings will be different from those of minimal SU(5), and the colored Higgs masses can be made sufficiently heavy that the proton decay constraints can be met [99]. Other possibilities for alleviating this problem include forbidding the dimension-five operator leading to proton decay using extra dimensions [103], more complicated Higgs sectors [104], flipped SU(5) [105–108], or a Peccei–Quinn symmetry [109]. The problem is also alleviated in models with scalar masses that are $\mathcal {O}(100)$ TeV [110–115], as in pure gravity mediation [116].

The LHC constraints on sfermion masses and the observed Higgs mass of 125 GeV motivate us to consider once again the decoupling limit as an explanation for the long lifetime of the proton. The decoupling limit was unsuccessful in [99] due to the assumption of a light third generation. However, if the third generation is also decoupled, the proton lifetime is extended. Since a heavier third generation is favored by the 125 GeV mass of the Higgs, we find this to be a reasonable scenario for suppressing proton decay. However, the real challenge in this scenario is to find regions of parameter space that combine a viable dark matter candidate with an acceptably long proton lifetime.

We will find that the minimal supersymmetric grand-unified theory based on SU(5) [117, 118] with a CMSSM spectrum does have a very small region of parameter space that combines a 125 GeV Higgs, a sufficiently long proton lifetime, and a viable dark matter candidate. This may occur either in the focus-point region [119–122] or in a region where the dark matter density is obtained by stop coannihilation with the bino [77, 123–127]. However, as we show below, sub-GUT and NUHM1 models are less restricted by proton decay constraints. The proton lifetime is longer in sub-GUT models, in general, because the stop masses are larger due to reduced RG running. Since the lifetime of the proton scales as a power of the stop mass, this enhances the proton lifetime. Moreover, these sub-GUT models have an acceptable dark matter density in regions where the bino and the lighter stau coannihilate [128–135]. In the NUHM1, $\sim$ TeV Higgsinos are possible for any value of $m_{0}$ and $m_{1/2}$ and, if the Higgsino mass is $\mathcal {O}$ (TeV), the Higgsino can be a thermal relic dark matter candidate [136, 137]. If $m_0$ and $m_{1/2}$ are large, the proton lifetime is greatly enhanced because of the large stop mass. In all cases, compatibility with minimal SU(5) requires relatively low values of $\tan \beta \lesssim 5$.

We also examine whether such models are compatible with present experimental constraints on the direct detection of dark matter through spin-independent elastic scattering, as provided, e.g., by the LUX experiment [138], and whether they can be probed by the next generation of such experiments, e.g., XENON1T [139] and LUX-ZEPLIN (LZ) [140].

For the purpose of our study, we use FeynHiggs [141–145] to calculate $m_{ h}$ as a function of the model input parameters. Since one expects an uncertainty $\sim \pm 1.5$ GeV in this calculation, we assume that any model yielding a prediction $m_{ h} \in [124, 127]$ GeV may be acceptable. Even with this theoretical uncertainty, we find that the $m_{ h}$ measurement generally gives a stronger constraint on the model parameters than do the direct LHC searches for supersymmetric particles published so far. As we also discuss, another important constraint is provided by the experimental search for $B_s \rightarrow \mu ^+ \mu ^-$ decay [146–149], particularly at large $\tan \beta$. Since we do not impose any $g_\mu - 2$ constraint, and the Higgs and other LHC constraints exclude small values of $(m_0, m_{1/2})$, the impact of the $b \rightarrow s \gamma$ constraint [150–153] is reduced.

We use the SuperSymmetry And Relic Density (SSARD) code (information as regards this code is available from K. A. Olive: it contains important contributions from J. Evans, T. Falk, A. Ferstl, G. Ganis, F. Luo, A. Mustafayev, J. Mc- Donald, K. A. Olive, P. Sandick, Y. Santoso, V. Spanos, and M. Srednicki). to calculate the particle spectrum, proton lifetime, and elastic scattering cross sections. SSARD first calculates the supersymmetric particle spectrum for a given set of boundary conditions defined by the model – CMSSM, NUHM etc. Coupled renormalization-group equations (RGEs) are then run back and forth between the weak scale and the GUT scale, which is defined by the renormalization scale where the two electroweak gauge couplings are equal. The gauge and Yukawa couplings are run at two loops, whereas the soft supersymmetry-breaking masses are run at one loop. The strong gauge coupling is fixed at the weak scale. The offset between the strong coupling and electroweak couplings is used to determine GUT-scale threshold corrections, and thereby the GUT-scale masses that affect proton decay. Once convergence of the RGEs is obtained, the sfermion mass matrices are evaluated to obtain physical masses. SSARD determines $\mu$ and $B \mu$ at the weak scale by minimizing the Higgs tadpole equations, and uses them to determine the pseudoscalar Higgs mass, which serves as an input to FeynHiggs. The neutralino and chargino mass matrices are then diagonalized with one-loop corrections applied. With the sparticle spectrum determined, the cross sections for annihilation and coannihilation are computed and input into a routine that integrates numerically the Boltzmann equation to determine the cosmological relic density. Finally, branching fractions for rare decays are computed, along with the value of $g_\mu -2$ and the neutralino–nucleon scattering cross section.

The layout of this paper is as follows. In Sect. 2 we summarize the features of the CMSSM and mSUGRA models that are relevant for our analysis, and introduce their extensions to sub-GUT and NUHM1 models. In Sect. 3, we discuss the basics of our calculations of the proton lifetime and elastic scattering cross sections in CMSSM-like models. Section 4 displays our results. Finally, Sect. 5 summarizes our conclusions.

## The models

### mSUGRA

Minimal supergravity (mSUGRA) models have a quadratic Kähler potential for the chiral superfields, and the effective scalar potential is [45, 154–160]

1 $$\begin{aligned} V= & {} \left| \frac{\partial W}{\partial \phi ^i}\right| ^2 + ( A_0 W^{(3)} + B_0 W^{(2)} + \text {h.c.}) + m_{3/2}^2 \phi ^i \phi _i^*,\nonumber \\ \end{aligned}$$

where *W* is the superpotential for the matter superfields $\phi _i$,^2^ which takes the following form in the MSSM:

2 $$\begin{aligned} W = \bigl ( y_e H_1 L \overline{e} + y_d H_1 Q \overline{d} + y_u H_2 Q \overline{u} \bigr ) + \mu H_1 H_2. \end{aligned}$$

We denote by *L* and *Q* (*e*, *u*, and *d*) the left- (right-)handed matter superfields, the Yukawa couplings are denoted by the $y_\alpha$, and $H_{1,2}$ are the pair of MSSM Higgs doublets with superpotential mixing coefficient $\mu$. The $W^{(3)}$ in Eq. (1) are the trilinear terms in the superpotential, $W^{(2)}$ is the bilinear part corresponding to the $\mu$ term, and $m_{3/2}$ is the gravitino mass. In mSUGRA one finds scalar mass universality with $m_0 = m_{3/2}$, and there is a relation between the tri- and bilinear supersymmetry-breaking terms:

3 $$\begin{aligned} A_0 = B_0 + m_0. \end{aligned}$$

These conditions apply at an input renormalization scale, $M_{\mathrm{in}}$, which may or may not be identified with the grand unification scale $M_{\mathrm{GUT}}$. If the gauge kinetic function is minimal, there is also gaugino mass universality, with a common mass $m_{1/2}$, which we assume to apply at the same input scale $M_{\mathrm{in}}$.

The two electroweak vacuum conditions are

4 $$\begin{aligned} \mu ^2=\frac{m_1^2-m_2^2\tan ^2\beta +\frac{1}{2}m_Z^2(1-\tan ^2\beta )+\Delta _{\mu }^{(1)}}{\tan ^2\beta -1+\Delta _{\mu }^{(2)}}\, \end{aligned}$$

and

5 $$\begin{aligned} B \mu = - \frac{1}{2}(m_1^2+m_2^2+2\mu ^2)\sin 2\beta +\Delta _B, \end{aligned}$$

where the soft supersymmetry-breaking Higgs masses denoted by $m_{1,2}$ are here evaluated at the weak scale, and $\Delta _B$ and $\Delta _\mu ^{(1,2)}$ are loop corrections [161–163]. An mSUGRA model has just three continuous parameters: $m_{1/2}$, $m_0$ and $A_0$. The conditions (4), (5) can be used to determine $\tan \beta$ as well as the magnitude of $\mu$, but the sign of $\mu$ is undetermined. We consider in this paper both signs of $\mu$ in selected cases.

### The CMSSM

The CMSSM is effectively a one-parameter generalization of mSUGRA, in which the relation (3) between $A_0$ and $B_0$ is dropped, which allows $\tan \beta$ to be taken as an extra free parameter. In addition, $m_0 \ne m_{3/2}$ in general, which is possible in SUGRA models only if the supergravity Kähler potential has non-minimal kinetic terms. Thus the CMSSM is specified by four parameters, $m_{1/2}$, $m_0$, $A_0$, $\tan \beta$, the sign of $\mu$. Here we assume that $m_{3/2}$ is sufficiently large to be irrelevant.

### Sub-GUT versions of the CMSSM and mSUGRA

Generalizations of both mSUGRA and the CMSSM are possible if the input scale for universality of the supersymmetry-breaking terms differs from $M_{\mathrm{GUT}}$. We concentrate here on ‘sub-GUT’ models with $M_{\mathrm{in}} < M_{\mathrm{GUT}}$ [39–41]: a value of $M_{\mathrm{in}}$ above the GUT scale [164–172] would introduce many more GUT parameters, requiring a separate in-depth study. Sub-GUT versions of mSUGRA have four parameters: $m_{1/2}, m_0 = m_{3/2}, A_0 = B_0 + m_0$, and $M_{\mathrm{in}}$, whereas sub-GUT versions of the CMSSM have $\tan \beta$ as an extra parameter (assuming again that $m_{3/2}$ is irrelevantly large). We found in [51] that sub-GUT mSUGRA models are phenomenologically viable in a relatively restricted range of $A_0$ that straddles the Polonyi value $A_0 = (3 -\sqrt{3}) \times m_{3/2}$ [45, 173].

### The NUHM1

Another one- or two-parameter generalization of the CMSSM is the NUHM [28, 29, 174–182], in which the values of the soft supersymmetry-breaking contributions to the Higgs masses $m_1$ and $m_2$ at the input scale are allowed to differ from the universal scalar mass $m_0$. In the NUHM1 considered here, it is assumed that $m_1 = m_2$ at the input scale [42–44]. One may choose either $\mu$ or $m_A$ (through its relation to $B\mu$) as a free parameter, and use the minimization conditions (4) and (5) to solve for $m_1 = m_2$. The examples shown here treat $\mu$ as a free parameter, since this displays more readily the interesting results in this scenario.

The two-parameter extension known as the NUHM2 [44, 183, 184] drops the requirement that $m_1 = m_2$ at the input scale. In this case, both $m_1$ and $m_2$ are allowed to be free input parameters. Alternatively, one can choose both $\mu$ and $m_A$ at the weak scale as free input parameters and use the minimization conditions (4) and (5) to solve for $m_1$ and $m_2$. We do not study the NUHM2 in this paper, as most of the interesting aspects of the NUHM2 are contained in NUHM1 scans.

## Calculations

### Proton decay lifetimes

In this subsection we describe how we calculate proton decay rates in the minimal supersymmetric SU(5) GUT model [117, 118]: for further discussions and detailed formulas, see [110, 115, 116, 185]. This model is the simplest supersymmetric extension of the original Georgi–Glashow model [186], in which the MSSM matter superfields are embedded into a $\overline{\mathbf{5}}\oplus \mathbf{10}$ representation of SU(5) for each generation, and the MSSM Higgs superfields $H_1$ and $H_2$ are incorporated in a pair of $\overline{\mathbf{5}}$ and **5** superfields, respectively. The SU(3)$_C$ components of the $\overline{\mathbf{5}}$ and **5** are called the color-triplet Higgs fields. The dominant contribution to proton decay in this model is given by the exchange of these color-triplet Higgs fields [187, 188], which induce dimension-five baryon-number violating operators, whereas the exchanges of SU(5) gauge bosons yield dimension-six operators. In this case, the dominant proton decay channel is the $p\rightarrow K^+\bar{\nu }$ mode, and we focus on the partial decay rate for this channel in the following.

We obtain the low-energy effective Lagrangian below the GUT scale by first integrating out the color-triplet Higgs fields. In superfield notation, the effective Lagrangian is given by

6 $$\begin{aligned} \mathcal{L}_5^\mathrm{eff}= C^{ijkl}_{5L}\mathcal{O}^{5L}_{ijkl} +C^{ijkl}_{5R}\mathcal{O}^{5R}_{ijkl} +\mathrm{h.c.}, \end{aligned}$$

with the effective operators $\mathcal{O}^{5L}_{ijkl}$ and $\mathcal{O}^{5R}_{ijkl}$ defined by

7 $$\begin{aligned} \mathcal{O}^{5L}_{ijkl}&\equiv \int \mathrm{d}^2\theta ~ \frac{1}{2}\epsilon _{abc} (Q^a_i\cdot Q^b_j)(Q_k^c\cdot L_l),\nonumber \\ \mathcal{O}^{5R}_{ijkl}&\equiv \int \mathrm{d}^2\theta ~ \epsilon ^{abc}\overline{u}_{ia}\overline{e}_j\overline{u}_{kb} \overline{d}_{lc}, \end{aligned}$$

where *a*, *b*, *c* are SU(3)$_C$ color indices, *i*, *j*, *k*, *l* are generation indices, and $\epsilon _{abc}$ is the totally antisymmetric tensor. The Wilson coefficients of the above operators, $C^{ijkl}_{5L}$ and $C^{ijkl}_{5R}$ at the GUT scale are evaluated from the tree-level color-triplet Higgs exchange diagrams, with the results given in Appendix A. As shown in Eq. (21) of the appendix, these Wilson coefficients include up-type quark Yukawa couplings $y_{u_i}$ and down-type quark/lepton Yukawa couplings $y_{d_l}$, which should be unified at the GUT scale in the minimal SU(5) GUT. Note, however, that although the minimal SU(5) GUT relation between the bottom and $\tau$ masses [189–192] is approximately consistent with the experimental values, this is not the case for the strange and $\mu$ masses, nor for the down and *e* masses. The GUT Higgs couplings must therefore be more complicated than in the minimal SU(5) GUT, e.g., with additional higher-dimensional Higgs representations [193] and/or contributions to the fermion masses from higher-dimensional superpotential terms [194]. In practice, the ambiguity in choosing whether the down-type quark or lepton Yukawa couplings sets the scale of proton decay results in about a factor of 20 uncertainty in the proton decay calculation [116], which represents our ignorance of GUT-scale physics in the Yukawa sector. In the following calculation, we take down-type quark Yukawa couplings as the GUT-scale Yukawa couplings, which leads to longer proton decay lifetimes and thus gives rather conservative bounds on the model parameter space.

In addition, the GUT-scale Yukawa couplings introduce two extra phase factors [195], which give rise to additional uncertainty [185]. It turns out, however, that the effects of these unknown phases are actually negligible, as shown in Appendix A. Thus, we neglect these effects in our analysis.

**Fig. 1:**
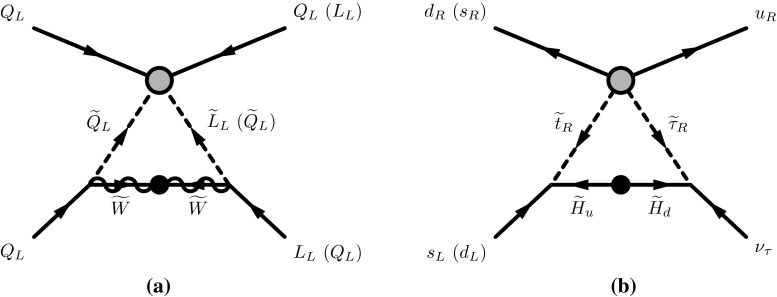
One-loop diagrams that yield dimension-six four-fermion operators. Diagrams **a** and **b** are generated by the charged wino and Higgsino exchange processes, respectively. The *gray dots* indicate the dimension-five effective interactions (6), and the *black dots* represent the wino and Higgsino mass terms

After integrating out the color-triplet Higgs boson, the GUT-scale Wilson coefficients are then evolved down to the sfermion mass scale according to the renormalization-group equations (RGEs), which are also presented in Appendix A. At the sfermion mass threshold, sfermions in the external lines of the dimension-five effective operators are integrated out via the one-loop diagrams illustrated in Fig. 1, yielding dimension-six four-fermion operators [196–198]. In the absence of flavor violation in the sfermion sector,^3^ only the operators, $\mathcal{O}^{5L}_{ii1j}$ and $\mathcal{O}^{5R}_{331k}$ with $i=2,3$, $j=1,2,3$, and $k =1,2$ give sizable contributions to proton decay; the contributions of the other operators are suppressed by small Yukawa couplings and/or the off-diagonal CKM matrix elements. The one-loop diagrams in Fig. 1 yield the following effective Lagrangian below the sfermion mass scale:

8 $$\begin{aligned} \mathcal{L}^{\text {eff}}_6&=C^{\widetilde{H}}_i \mathcal{O}_{1i33} + C^{\widetilde{W}}_{jk}\widetilde{\mathcal{O}}_{1jjk} + C^{\widetilde{W}}_{jk}\widetilde{\mathcal{O}}_{j1jk} + \overline{C}^{\widetilde{W}}_{jk}\widetilde{\mathcal{O}}_{jj1k}, \end{aligned}$$

with the operators composed of SM fermion fields,

9 $$\begin{aligned} \mathcal{O}_{ijkl}&\equiv \epsilon _{abc}(u^a_{Ri}d^b_{Rj}) (Q_{Lk}^c \cdot L_{Ll}), \nonumber \\ \widetilde{\mathcal{O}}_{ijkl}&\equiv \epsilon _{abc} \epsilon ^{\alpha \beta } \epsilon ^{\gamma \delta } (Q^a_{Li\alpha }Q^b_{Lj\gamma }) (Q_{Lk\delta }^c L_{Ll\beta }) , \end{aligned}$$

corresponding, respectively, to the operators $O^{(1)}$ and $\widetilde{O}^{(4)}$ in [198], where $\alpha ,\beta ,\gamma ,\delta$ are SU(2)$_L$ indices, $\epsilon ^{\alpha \beta }$ is the totally antisymmetric tensor, and $i =1,2$, $j=2,3$, and $k=1,2,3$. The coefficients of the operators in (8) are also given in Appendix A. Note that, since a chirality flip in the internal wino/Higgsino propagator is required in the processes shown in Fig. 1, the operator coefficients $C^{\widetilde{H}}_i, C^{\widetilde{W}}_{jk}$ contain factors of $M/M_{\widetilde{f}}^2$ if $M\lesssim M_{\widetilde{f}}$, where *M* is the wino or Higgsino mass and $M_{\widetilde{f}}$ is the mass of a sfermion running in the loop. As a result, if the magnitude of the Higgsino mixing term $|\mu |$ is much smaller than that of the wino mass $|M_2|$, the wino contribution dominates the Higgsino contribution, and vice versa if $|\mu | \gg |M_2|$.

The coefficients in (8) are then run down to the electroweak scale using the one-loop RGEs given in [199]. At the electroweak scale, we transform to an operator basis in the low-energy $\text {SU}(3)_C\otimes \text {U}(1)_{\text {em}}$ theory, and the operator coefficients are evolved to the hadron scale, $Q_{\text {had}} =2$ GeV, using the two-loop RGEs obtained in [200]. Finally, using the hadron matrix elements of the operators at $Q_{\text {had}} =2$ GeV, we obtain the partial decay width of the $p\rightarrow K^+ \bar{\nu }$ channel. These matrix elements are evaluated using the QCD lattice simulation performed in [201]. This procedure, as well as the relevant formulas, is also summarized in Appendix A.

As can be seen from (21), the proton decay rate depends on the mass of the color-triplet Higgs field $M_{H_C}$. Thus, to evaluate the proton lifetime, we need to determine the size of $M_{H_C}$. To that end, we use the method discussed in [100–102]. In this method, the GUT-scale threshold corrections to the gauge coupling constants are used to estimate the masses of the GUT particles. The GUT-scale matching conditions for the gauge coupling constants at one-loop level in the $\overline{\mathrm{DR}}$ scheme [202] in the minimal SU(5) GUT are given as follows [203–205], assuming no additional GUT-scale physics:

10 $$\begin{aligned} \frac{1}{g_1^2(M_{\mathrm{GUT}})}&=\frac{1}{g_5^2(M_{\mathrm{GUT}})}+\frac{1}{8\pi ^2}\biggl [ \frac{2}{5} \ln \frac{M_{\mathrm{GUT}}}{M_{H_C}}\nonumber \\&\quad -10\ln \frac{M_{\mathrm{GUT}}}{M_X} \biggr ],\nonumber \\ \frac{1}{g_2^2(M_{\mathrm{GUT}})}&=\frac{1}{g_5^2(M_{\mathrm{GUT}})} +\frac{1}{8\pi ^2}\biggl [ 2\ln \frac{M_{\mathrm{GUT}}}{M_\Sigma }\nonumber \\&\quad -6\ln \frac{M_{\mathrm{GUT}}}{M_X} \biggr ],\nonumber \\ \frac{1}{g_3^2(M_{\mathrm{GUT}})}&=\frac{1}{g_5^2(M_{\mathrm{GUT}})} +\frac{1}{8\pi ^2}\biggl [ \ln \frac{M_{\mathrm{GUT}}}{M_{H_C}}\nonumber \\&\quad +3\ln \frac{M_{\mathrm{GUT}}}{M_\Sigma }-4\ln \frac{M_{\mathrm{GUT}}}{M_X} \biggr ], \end{aligned}$$

where $g_1$, $g_2$, $g_3$, and $g_5$ are the gauge coupling constants of U(1), SU(2)$_L$, SU(3)$_C$, and SU(5), respectively, with $g_1$ related to the hypercharge gauge coupling $g^\prime$ through $g_1 = g^\prime \sqrt{5/3}$, and $M_X$ and $M_\Sigma$ are the masses of the heavy gauge bosons and the adjoint Higgs fields, respectively. Note that these conditions do not include scale-independent terms since we use the $\overline{\mathrm{DR}}$ scheme for the renormalization. These equations then yield

11 $$\begin{aligned}&\frac{3}{g_2^2(M_{\mathrm{GUT}})}- \frac{2}{g_3^2(M_{\mathrm{GUT}})} - \frac{1}{g_1^2(M_{\mathrm{GUT}})} \nonumber \\&\quad =-\frac{3}{10\pi ^2}\ln \biggl (\frac{M_\mathrm{GUT}}{M_{H_C}}\biggr ), \nonumber \\&\frac{5}{g_1^2(M_{\mathrm{GUT}})}- \frac{3}{g_2^2(M_{\mathrm{GUT}})} - \frac{2}{g_3^2(M_{\mathrm{GUT}})} \nonumber \\&\quad =-\frac{3}{2\pi ^2}\ln \biggl ( \frac{M_{\mathrm{GUT}}^3}{M_X^2M_{\Sigma }}\biggr ), \end{aligned}$$

and the upper relation allows one to evaluate $M_{H_C}$ from the coupling constants of the SM gauge interactions at the GUT scale determined using the RGEs [100–102].

Before concluding this subsection, we discuss the qualitative dependence of the proton decay lifetime on the MSSM parameters. As already mentioned above, the loop functions for the diagrams in Fig. 1 give rise to a factor of $\sim M/M_{\widetilde{f}}^2$. Therefore, the proton lifetime becomes longer if the sfermion masses are taken to be larger. In addition, as can be seen from (21), the decay amplitude contains both the up- and down-type Yukawa couplings, which leads to a factor of $1/\sin 2\beta$. Moreover, the Higgsino exchange contribution also has an extra factor of $1/\sin 2\beta$. As a result, the proton decay rate is strongly enhanced for moderate/large values of $\tan \beta$. For these reasons, large sfermion masses and small $\tan \beta$ are favorable for evading the proton decay constraints.

### Elastic scattering cross sections

Next, we review the calculation of the neutralino–nucleus elastic scattering cross sections that we use in the following analysis. There are two types of interactions that induce dark matter-nuclei scattering: spin-independent (SI) and spin-dependent (SD). Since there is no interference between these two interactions, we can evaluate the SD and SI scattering cross sections separately.

We first consider SI scattering. The SI elastic scattering cross section of the neutralino lightest supersymmetric particle (LSP) with a nucleus is expressed in terms of the SI neutralino–nucleon effective coupling $f_N$ ($N=p, n$) as follows:

12 $$\begin{aligned} \sigma _{\text {SI}} = \frac{4}{\pi }\left( \frac{m_\chi m_T}{m_\chi + m_T}\right) ^2 \left[ Z f_p +(A-Z) f_n\right] ^2, \end{aligned}$$

where $m_\chi$ and $m_T$ are the masses of the neutralino LSP and the target nucleus, respectively, and *Z* (*A*) denotes the atomic (mass) number of the target nucleus.

The SI neutralino–nucleon scattering matrix elements are induced by the exchange of squarks and neutral Higgs bosons. To evaluate the effective coupling $f_N$, we first obtain the neutralino–quark/gluon effective operators by integrating out the squarks and Higgs bosons. Then, using the nucleon matrix elements of these effective operators, we can calculate the effective coupling $f_N$. For more details, see [206–214]. As a result, $f_N$ is expressed in terms of the coefficients of the neutralino–quark effective scalar interactions, $\alpha _{3q} \overline{\chi }\chi \overline{q}q$ (where $\chi$ and *q* denote the neutralino LSP and quarks, respectively), as [208–212]

13 $$\begin{aligned} \frac{f_N}{m_N} = \sum _{q=u,d,s} f^{(N)}_{T_q} \frac{\alpha _{3q}}{m_q} +\frac{2}{27}f^{(N)}_{TG} \sum _{q=c,b,t}\frac{\alpha _{3q}}{m_q}. \end{aligned}$$

Here the $m_q$ are the quark masses, $m_N$ is the nucleon mass, the $f_{T_q}^{(N)} \equiv \langle N| m_q \overline{q} q |N\rangle / m_N$ are the nucleon matrix elements of the light-quark mass operators, and $f_{TG}^{(N)} \equiv 1- \sum _{q=u,d,s} f_{T_q}^{(N)}$ denotes the gluon contribution to the nucleon mass. We extract the values of $f_{T_q}^{(N)}$ from the pion–nucleon $\sigma$-term $\Sigma _{\pi N}=50$ and $\sigma _0 = 36$ MeV [215].^4^ Analytic expressions for the $\alpha _{3q}$ are presented in [208–212]. The second term of the right-hand side in Eq. (13) represents the (long-distance) contribution of heavy quarks to the neutralino–gluon interactions, which can be related to the quark couplings $\alpha _{3q}$ via the triangle diagrams associated with the trace anomaly of the energy-momentum tensor [223, 224]. In Eq. (13), we neglect the effects of the twist-2 operators [206] as well as the short-distance contribution of quarks to the gluon operators [213, 214], since they are numerically small when squarks are rather heavy, which is the case we discuss below.

In the models we study, the dominant contribution to $\alpha _{3q}$ is given by the exchange of neutral Higgs bosons, since the squarks tend to be heavy. Moreover, in a wide range of parameter space, the Higgs sector is close to the decoupling limit, and the LSP is a bino–Higgsino mixed state with $|M_1|$, $|\mu |$, $|M_1-|\mu || \gg m_Z$. In this case, the expression for $\alpha _{3q}$ is approximated by

14 $$\begin{aligned} \alpha _{3q} \simeq - \frac{g_2^2 m_q \tan ^2 \theta _W}{4(\mu ^2-M_1^2)} \left( \frac{M_1+\mu \sin 2\beta }{m_h^2} +\frac{\mu \cos 2\beta }{m_H^2} c_q \right) , \end{aligned}$$

for the bino LSP case, while

15 $$\begin{aligned} \alpha _{3q} \simeq -\frac{\text {sgn}(\mu )g_2^2m_q}{8}\left( \frac{\tan ^2 \theta _W}{M_1-|\mu |}+\frac{1}{M_2-|\mu |}\right) \nonumber \\ \left[ \frac{1+\text {sgn}(\mu )\sin 2\beta }{m_h^2}+ \frac{\text {sgn}(\mu )\cos 2\beta }{m_H^2}c_q\right] , \end{aligned}$$

for the Higgsino LSP case. Here, $\theta _W$ is the Weinberg mixing angle, $m_h$ ($m_H$) is the mass of the lighter (heavier) neutral Higgs boson, and $c_q = \cot \beta$ and $-\tan \beta$ for up- and down-type quarks, respectively. As can be seen from these expressions, the neutralino–nucleon scattering cross sections decrease when the difference between $M_1$ and $|\mu |$ gets large. In addition, it is found that the SI effective coupling depends on the sign of $\mu$ and, in particular, when $\mu$ is negative the coupling can be significantly suppressed due to cancellations (this feature is sometimes called the “blind spot” [208–211, 222, 225–228].

We next discuss SD scattering, for which the neutralino–nucleus scattering cross section is given by

16 $$\begin{aligned} \sigma _{\text {SD}}= \frac{32}{\pi }G_F^2\Lambda ^2 J (J+1)\left( \frac{m_\chi m_T}{m_\chi + m_T}\right) ^2, \end{aligned}$$

where $G_F$ is the Fermi constant, *J* is the total spin of the target nucleus, and

17 $$\begin{aligned} \Lambda = \frac{1}{J}(a_p \langle S_p \rangle + a_n \langle S_n \rangle ), \end{aligned}$$

with $\langle S_p \rangle$ ($\langle S_n \rangle$) being the expectation value of the total spin of protons (neutrons) in the target nucleus. The SD neutralino–nucleon effective coupling, $\alpha _N$, is expressed as

18 $$\begin{aligned} a_N =\sum _{q=u,d,s} \frac{\alpha _{2q}}{\sqrt{2} G_F} \Delta ^{(N)}_q, \end{aligned}$$

where we use $\Delta ^{(N)}_q$ given in [212], and $\alpha _{2q}$ denotes the SD neutralino–quark couplings, which are induced by the exchange of *Z*-boson and squarks. The analytic formula for $\alpha _{2q}$ is again given in [208–212]. As in the SI case, the squark contribution is suppressed compared with the *Z* boson contribution in the parameter region we are interested in. Furthermore, when $|M_1|$, $|\mu |$, $|M_1-|\mu || \gg m_Z$, $a_{2q}$ is approximated by

19 $$\begin{aligned} a_{2q} \simeq \frac{g_2^2\tan ^2 \theta _W}{8 (\mu ^2-M_1^2)} \cos 2\beta T_q^3, \end{aligned}$$

for a bino-like LSP, while for a Higgsino-like LSP we have

20 $$\begin{aligned} \alpha _{2q}\simeq \frac{g_2^2}{16|\mu |} \cos 2\beta T_q^3 \left( \frac{\tan ^2\theta _W}{M_1}+\frac{1}{M_2} \right) , \end{aligned}$$

where $T_q^3$ denotes the third component of the SU(2)$_L$ generators.

As seen above, the neutralino–nucleus scattering cross sections are suppressed when gauginos/Higgsinos are heavy. In such cases, electroweak loop contributions may dominate the tree-level Higgs and *Z* boson contributions [213, 229–232]. It turns out, however, that in the case of a bino–Higgsino LSP, the electroweak loop contributions are quite small [233], and thus we neglect them in our calculation.

Because of the coherent nature of the SI neutralino–nucleus scattering as shown in Eq. (12), the current and future direct detection experiments are much more sensitive to the SI scattering compared to SD scattering. For this reason, we mainly discuss SI scattering in the following.

## Results

### CMSSM

**Fig. 2:**
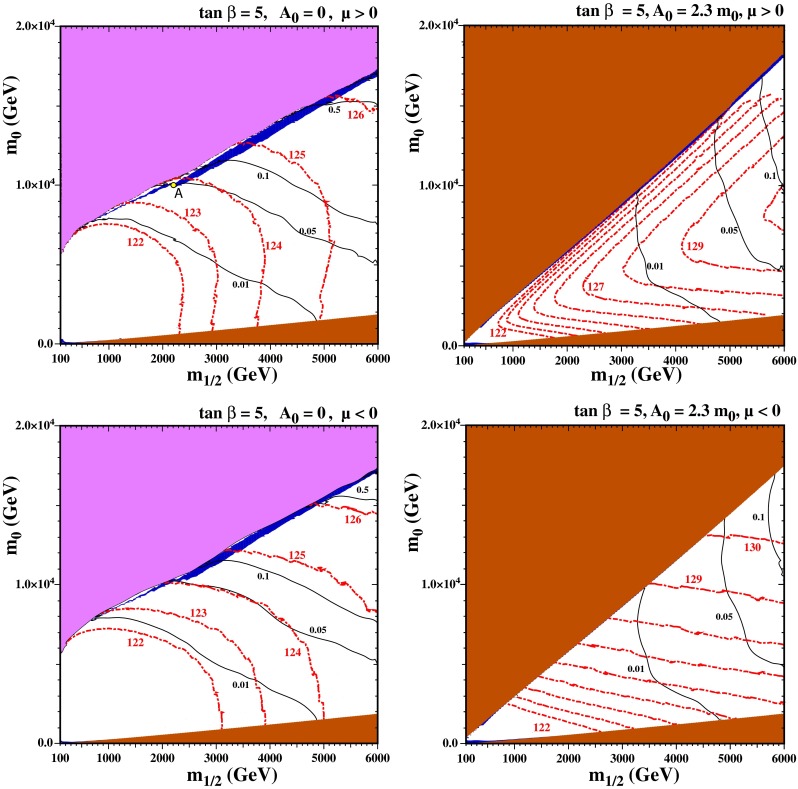
The CMSSM $(m_{1/2}, m_0)$ planes for $\tan \beta = 5$ with $\mu > 0$ (*upper*) and $\mu < 0$ (*lower*), and with and $A_0 = 0$ (*left*) and $A_0 = 2.3 \, m_0$ (*right*). In the *light mauve shaded regions*, it is not possible to satisfy the electroweak symmetry-breaking (EWSB) conditions. In the *brown shaded regions*, the LSP is charged and/or colored. The *dark blue shaded regions* show the areas where $0.06 < \Omega _\chi h^2 < 0.2$ in the *left panels* and the further enlarged range of $0.02 < \Omega _\chi h^2 < 0.5$ in the *right panels*. The *red dot-dashed contours* indicate the Higgs mass, labeled in GeV, and the *solid black contours* indicate the proton lifetime in units of $10^{35}$ years. The *point labeled A* refers to the point tested for phase dependence in the appendix

In view of the discussion in Sect. 3.1, in our study of the proton lifetime we focus on relatively small values of $\tan \beta$, and have chosen $\tan \beta = 5$ in Fig. 2. For larger values of $\tan \beta$, the proton lifetime becomes smaller than the current experimental bound, and minimal SU(5) is not viable.

We show in Fig. 2 four examples of $(m_{1/2}, m_0)$ planes in the CMSSM with $\tan \beta = 5$. In the left panels we choose $A_0 = 0$, whereas in the right panels we choose $A_0 = 2.3 \, m_0$. We take $\mu > 0$ in the upper panels and $\mu < 0$ in the lower panels. Higgs mass contours are shown as red dot-dashed curves labeled by $m_h$ in GeV in 1 GeV intervals starting at 122 GeV. We recall that to calculate $m_h$ we use FeynHiggs [141–145], which carries a roughly $\pm$1.5 GeV uncertainty. In the left panels, the light mauve shaded region in the upper part of the figure is excluded because there are no solutions to the Higgs minimization conditions: along this boundary $\mu ^2 = 0$. Electroweak symmetry breaking (EWSB) fails here because the Higgs soft masses at the GUT scale are large and the RG running to the weak scale does not suppress the Higgs soft masses sufficiently for EWSB to occur. Because large gluino masses can assist electroweak symmetry breaking effectively at two loops, the value of $m_0$ that is allowed increases for increasing $m_{1/2}$. Just below the region where EWSB fails, there is a dark blue shaded region where the relic density falls within the range determined by CMB experiments [234]. Since the relic density of dark matter is now determined quite accurately ($\Omega _\chi h^2 = 0.1193 \pm 0.0014$), for the purpose of visibility we show in dark blue the strip for which the relic density lies in the range [0.06, 0.20]. This strip is in the focus-point region [75, 76, 119–122] where the Higgsinos are much lighter than the stops. The correct dark matter density is realized either by coannihilation of the Higgsino with the bino when $m_{1/2}$ is smaller, or by Higgsino annihilations when the Higgsino mass is of order a TeV for larger values of $m_{1/2}$. The TeV-scale Higgsino dark matter region continues well beyond the bounds of the figure.

We note also that the brown shaded regions at the bottoms of the panels are excluded because there the LSP is the lighter charged stau lepton. The planes also feature stau coannihilation strips close to the boundary of the brown shaded region. They extend to about $m_{1/2} \simeq 1$ TeV but are very difficult to see on the scale of this plot, even with our enhancement of the relic density range. We note that for this value of $\tan \beta$ there are no relevant constraints from rare B decays.

Contours of the proton lifetime using down-type Yukawa couplings (see the discussion given in Sect. 3.1) are shown as solid black curves that are labeled in units of $10^{35}$ years. Thus the limit of $\tau _p > 5 \times 10^{33}$ years would exclude everything below the curve labeled 0.05. For the nominal value of $m_h = 125$ GeV, neglecting the theoretical uncertainties in the calculation of $m_h$, we see that in the upper left plane of Fig. 2 the Higgs contour intersects the focus-point region where $\tau _p \approx 2 \times 10^{34}$ years. Much of the focus-point strip in this figure may be probed by future proton decay experiments. Changing the sign of $\mu$ has almost no effect on the proton lifetime, as seen in the lower left panel of Fig. 2, but the calculated Higgs mass is smaller by $\sim$1 GeV, which is less than the uncertainty in the FeynHiggs calculation of $m_h$.

In the right panels of Fig. 2, since increasing $A_0$ drives a larger splitting between the two stops, there are excluded regions shaded in brown in the upper halves of the panels, where the lighter stop becomes the LSP. Close to this boundary (but again difficult to see) there is a narrow blue strip where $0.02 < \Omega _\chi h^2 < 0.5$. Here the dark matter density is brought into the allowed range by coannihilation with the lighter stop. The relatively large value of $A_0 = 2.3 \, m_0$ leads to large Higgs masses in most of the plane, but the Higgs mass is somewhat smaller along this strip for $\mu > 0$. We recall that the Higgs mass is sensitive to the off-diagonal element in the stop mass matrix, which is proportional to $X_t = A_t + \mu \cot \beta$ and peaks when $X_t$ is roughly 2.5 times the geometric mean of the two stop masses. For positive $\mu$, $X_t$ is relatively large and we are past the peak where $m_h$ is maximized. In contrast, for $\mu < 0$, $X_t$ is smaller (as there is some cancellation between the two terms) and we are closer to the peak and $m_h$ is larger. This effect is pronounced along the upper left edge because the stop is much lighter in this region. For $\mu > 0$, the $m_h = 124$ GeV contour, which is consistent with the experimental value when uncertainties in the theoretical calculation are considered, intersects the strip at $m_{1/2} \sim 4.7$ TeV where $\tau _p = 5 \times 10^{33}$ years. Note that we terminate the larger $m_h$ contours where the calculated value becomes unreliable: near the endpoints of these curves, the uncertainty in the FeynHiggs calculation of $m_h$ is $\gtrsim 5$ GeV. For $\mu < 0$, the $m_h = 125$ GeV contour intersects the stop coannihilation strip when $m_{1/2} \sim 1.4$ TeV and the proton lifetime is significantly smaller ($<$0.001 in these units). For slightly lower $A_0$ than the value $2.3 \, m_0$ shown in these panels of Fig. 2, large uncertainties in $m_h$ from FeynHiggs appear when $\tau _p < 5 \times 10^{33}$ years. When $A_0/m_0 \lesssim 2.0$ the stop coannihilation strip is no longer present. On the other hand, when $A_0/m_0 \gtrsim 2.4$ the central value of the Higgs mass along the stop strip drops below 122 GeV when $\mu > 0$, which is unacceptably small.

**Fig. 3:**
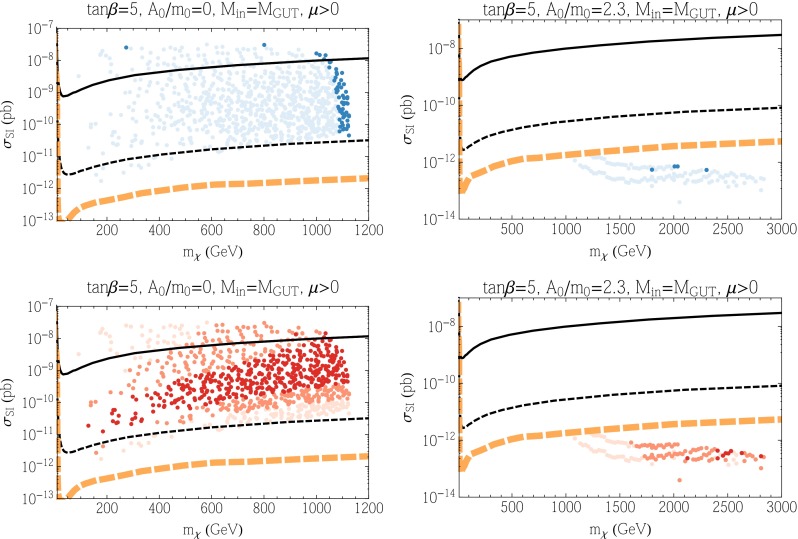
The spin-independent elastic scattering cross section in the CMSSM as a function of the neutralino mass for $\mu > 0$, with $\tan \beta = 5$ and $A_0 = 0$ (*left*) and $A_0 = 2.3 \, m_0$ (*right*). The *upper panels* show points where the relic density is within 3$\sigma$ of the central Planck value *colored darker blue*, and those where the relic density is below the Planck value as *lighter blue points*. The *lower panels* show the same set of points colored according to the calculation of the Higgs mass: 124–126 GeV (*darkest*), 123–124 and 126–127 GeV (*lighter*), 122–123 and 127–128 GeV (*lightest*). The *black solid curve* is the current LUX bound. The *black dashed curve* is the projected LZ sensitivity and the *dashed orange curve* is the neutrino background level

We show in Fig. 3 the spin-independent cross section, $\sigma _\mathrm{SI}$, as a function of the neutralino mass for the two upper panels in Fig. 2 with $\mu > 0$. The points in each panel represent results of a scan of the parameter space. In the upper panels, darker points fall within 3$\sigma$ of the dark matter relic density that fits best the Planck data. Lighter points have smaller relic densities and should not be excluded. However, whenever the relic density is below the central value determined by Planck, we scale the cross section downward by the ratio of the calculated density to the Planck density. From these panels, we find that the $A_0 = 0$ cases give relatively large SI scattering cross sections, while those for $A_0 = 2.3m_0$ are significantly suppressed. In the case of $A_0 = 0$, the values of $\mu$ and $M_1$ are close to each other, and thus the LSP is a well-mixed bino–Higgsino state. This leads to a large SI scattering cross section, as can be seen from Eqs. (14) and (15). The set of darkly shaded points with good relic density are found mostly at $m_\chi \simeq 1100$ GeV due to the fact that these points are mainly Higgsino LSPs. As the bino mass is increased, the scattering cross section decreases. However, the points sampled here all have $m_h < 128$ GeV which produces the lower boundary of the points displayed. Because of the constraints coming from the Higgs mass, the scattering cross sections for all dark matter candidates in these models are accessible at LZ. Along the focus point, the LSP mass varies downward as $\mu$ is decreased and the cross section is maximal at around $3 \times 10^{-8}$ pb. Due to the small uncertainty in the Planck relic density, we find very few darkly shaded points in this region. Points with smaller cross section are found between the focus-point strip and the no-EWSB boundary where the relic density is below the Planck density. On the other hand, for the $A_0 = 2.3m_0$ cases, the LSP is almost pure bino and squarks are quite heavy; for these reasons, we obtain very small SI cross sections. The solid curve in Fig. 3 corresponds to the current LUX limit [138] and thus some models (including the focus-point models) are excluded by this limit when $A_0 = 0$, assuming our nominal treatment of $\sigma _\mathrm{SI}$ as discussed in Sect. 3.2. The thin black dashed curve corresponds to the projected LZ sensitivity [140, 235] and almost all of the points sampled when $A_0 = 0$ are therefore testable. The thick orange dashed line corresponds to the irreducible neutrino background [235, 236]. All of the points sampled when $A_0 = 2.3 m_0$ fall below the neutrino background and probing them would require a directional recoil detector [237].

In the lower panels of Fig. 3, we see the same points, now colored to show the Higgs mass ranges. The darkest points have the calculated Higgs mass in the range 124–126 GeV, medium shaded points have $m_h$ in the ranges 123–124 or 126–127 GeV, and the lightest points have $m_h$ in the ranges 122–123 or 127–128 GeV. All of these are compatible with the experimental measurement, within twice the FeynHiggs uncertainty.

**Fig. 4:**
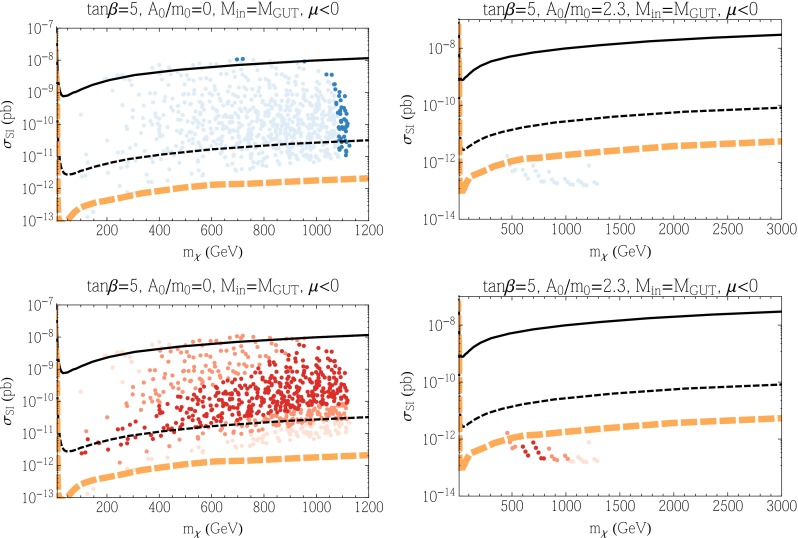
As in Fig. 3, but for $\mu < 0$

In Fig. 4, we show the points corresponding to the lower panels of Fig. 2 with $\mu < 0$. There is relatively little change in the scattering cross sections for $\mu <0$. When $A_0 = 0$, the cross sections are in general somewhat lower due to the cancellation mentioned in Sect. 3.2. For $A_0 = 2.3m_0$ the points have moved to lower $m_\chi$, but they remain for the most part unobservable. We again see that the Higgs mass constraint puts the bulk of the points within reach of LZ.

**Fig. 5:**
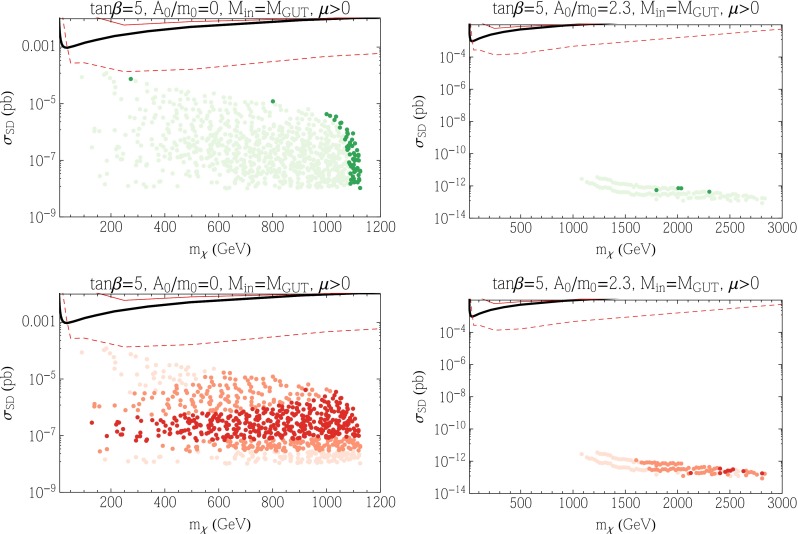
As in Fig. 3, but showing the spin-dependent cross section. The *solid curve* is the current PICO bound [238], and the *red solid/dashed curves* are the IceCube bounds [239] assuming annihilations into ${\bar{b}}b/W^+W^-$, respectively. The *upper panels* show points where the relic density is within 3$\sigma$ of the central Planck value colored *darker green*, and those where the relic density is below the Planck value as *lighter green points*. The *color coding* of the *lower panels* are the same as in Fig. 3

Finally, in Fig. 5 we show the spin-dependent cross sections, $\sigma _\mathrm{SD}$, for the upper panels in Fig. 2 when $\mu > 0$. Points where the relic density is within 3$\sigma$ of the central Planck value are colored darker green in the upper panels, and those where the relic density is below the Planck value as lighter green points, and the other points and shadings are identical to those in the previous two figures. Here the thick black solid curve is the upper limit from PICO [238] and the thin curves are obtained from IceCube [239] limits based on annihilations into $b {\bar{b}}$ pairs (solid) or $W^+ W^-$ pairs (dashed). For the focus-point models, annihilations proceed primarily into electroweak gauge bosons, or *hZ* final states with some non-negligible contributions from $t \bar{t}$, for which the $W^+ W^-$ may be applicable. Models with $A_0 = 0$ lie just below the current bounds again because of the highly mixed nature of the LSP, while the models with $A_0 = 2.3 m_0$ predict cross section far below these bounds.

### mSUGRA

In [51], mSUGRA models were considered with $A_0/m_0 = 3-\sqrt{3}$ (the Polonyi [173] value) and $A_0/m_0 = 2$ for comparison. The computed value of $\tan \beta$ is generally $\gtrsim 10$. As a result, proton lifetime limits for these cases would be short, in violation of the experimental bounds unless a non-minimal version of SU(5) is adopted.

In the case of the Polonyi model, viable regions of the parameter space contain a gravitino LSP, which would give negligible signals in direct detection experiments. However, for the larger value $A_0 = 2 m_0$ there are some regions of parameter space with a bino LSP with the relic density held in check by stau coannihilations, but $\tan \beta \gtrsim 40$ for $m_h > 124$ GeV and there are strong constraints from B-physics observables in that case. This model is highly constrained and we do not discuss it further here, though we return later to mSUGRA models with universality imposed below the GUT scale.

### subGUT

**Fig. 6:**
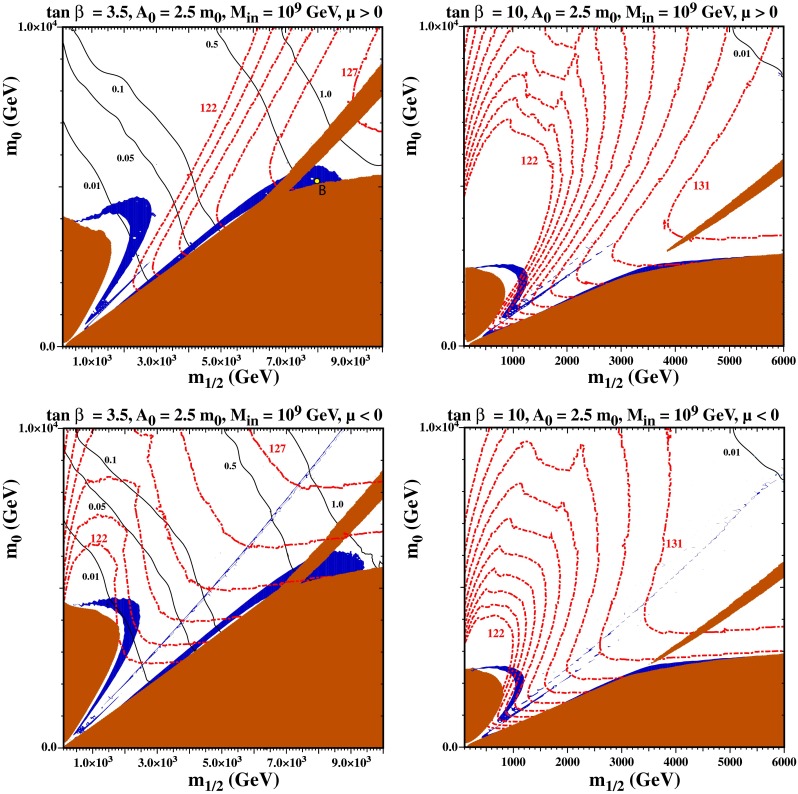
The subGUT CMSSM $(m_{1/2}, m_0)$ planes for $A_0 = 2.5 \, m_0$ with $M_{\mathrm{in}} = 10^9$ GeV. The *left* (*right*) *panels* have $\tan \beta = 3.5 (10)$. The *upper* (*lower*) *panels* have $\mu > 0$ ($\mu < 0$). The *shadings* and *contour* types are as in Fig. 2. The *point labeled B* refers to the point tested for phase dependence in the appendix

In the left panels of Fig. 6, we show examples of $(m_{1/2}, m_0)$ planes with $\tan \beta = 3.5$, $A_0 = 2.5 \, m_0$, $M_{in} = 10^9$ GeV and $\mu > 0$ (upper panel) and $\mu < 0$ (lower panel). In both planes, one finds three distinct brown shaded regions where the LSP is no longer neutral and/or uncolored. At the left, at low $m_{1/2}$ and $m_0 < 4$ TeV the lighter stop is the LSP, at low $m_0$ for all $m_{1/2}$ the lighter stau is the LSP, and along a diagonal strip that rises from the stau LSP region the lighter chargino is the LSP. When $\mu$ is approximately equal to the bino mass, $M_1$, two of the neutralino mass eigenstates are strongly mixed bino–Higgsino states. In this case, 1-loop corrections to these masses can differ significantly from the correction to the second Higgsino (which is nearly identical to the correction to the lighter chargino) and cause the chargino to become the LSP. To the left of this region, the LSP is the bino and the relic density gets too large. To the right of this region, the LSP is the Higgsino and, because $m_{1/2}$ and $\mu$ are large here, the relic density again gets too large.

There are also three distinct regions in these panels of Fig. 6 where the relic density is consistent with the Planck constraint. Somewhat offset from the stop LSP region, we see a curved band which is produced by stop coannihilation. This region is much broader than in the typical CMSSM case, due to the increased degeneracy of the SUSY particles. Then, just above the stau LSP region we see the familiar stau coannihilation strip. At this value of $M_{\mathrm{in}}$ it extends to far greater values of $m_{1/2}$ than it would in the CMSSM with $M_{\mathrm{in}} = M_{\mathrm{GUT}}$: this is generally possible for sufficiently small $M_{\mathrm{in}}$, and is again due to the degeneracy of the SUSY particles. We note that, to the right of the chargino LSP region, stau coannihilation occurs between the stau and a Higgsino LSP instead of the more usual bino. Finally, between the stau and stop strips, we see a narrow funnel region where $2 m_\chi \approx m_{A,H}$. For $\mu > 0$, the funnel extends to $m_{1/2}, m_0 \lesssim 3$ TeV, while for $\mu < 0$, it extends past the end of the plot to $m_0 > 10$ TeV. Looking now at the red dashed contours of $m_h$, we see that in the stop coannihilation strip and the funnel region the Higgs mass is somewhat too small: $m_h < 123$ GeV for $\mu > 0$, whereas the uncertainty from FeynHiggs is $\sim$1.5 GeV. This is to be expected, since the stop masses are light in this region and so the corrections to the Higgs mass are small. On the other hand, the Higgs mass exceeds 124 GeV for $4~\mathrm{TeV} < m_{1/2}$, and hence much of the stau coannihilation region is acceptable. For $\mu < 0$ (lower left panel of Fig. 6), the values of $m_h$ are somewhat higher, and parts of the stop coannihilation strip may be acceptable.

As in the previous subsections, the solid black lines are contours of the proton decay lifetime. For $4~\mathrm{TeV} < m_{1/2}$ (where the Higgs mass is acceptable), the lifetime exceeds the experimental bound of $5 \times 10^{33}$ years. As discussed earlier, in order to obtain a sufficiently long lifetime, we are forced to relatively small values of $\tan \beta$. For $\tan \beta$ larger than the value 3.5 shown here, the lifetime along the stau strip drops below the experimental bound, as seen in the right panels of Fig. 6 where $\tan \beta = 10$ is chosen, and one would need to abandon minimal SU(5), as the proton lifetime is less than $10^{33}$ years over much of the plane.

Qualitatively, we see similar features for the LSP and relic density in the right panels. Smaller $$\tan \beta $$ ($$< $$3.5) is possible, but one needs to go to higher values of $$m_{1/2}$$ to ensure a sufficiently heavy Higgs boson. For smaller $$A_0/m_0$$, the extent of the stau strip is reduced, making it difficult to obtain both a heavy enough Higgs and a long proton lifetime. This reduction in the stau coannihilation strip is due to a reduction in the Higgsino masses as $$A_0$$ is reduced. The stau coannihilation band in this figure is actually assisted by several other supersymmetric particles with masses similar to the stau, including the charged and neutral Higgsinos. The Higgsino masses are roughly set by $$m_0$$ ($$\mu $$ is set by EWSB conditions which is related to the stop mass and hence to $$m_0$$). Since the bino and stau masses continue to grow as $$m_{1/2}$$ is increased, eventually the Higgsino becomes the LSP with no potential coannihilation partners. At this point, the stau coannihilation band disappears and the Higgsino becomes the LSP. Lowering $$A_0$$ reduces the value of $$m_0$$ for which the Higgsino becomes the LSP, and so reduces the size of the stau coannihilation strip. However, if we rely on non-minimal SU(5) to lengthen the lifetime of the proton, we can go to larger values of $$\tan \beta $$, as seen in the right panels of Fig. 6. This allows one to obtain simultaneously a large enough Higgs mass and a small enough relic density.

**Fig. 7:**
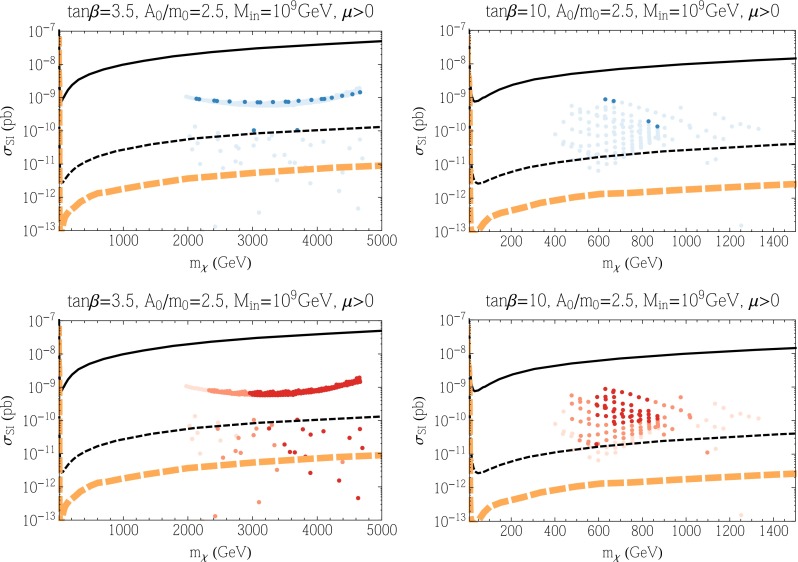
As in Fig. 3 for the subGUT case with $A_0 = 2.5 m_0$, $\mu > 0$, $M_{\mathrm{in}} = 10^9$ GeV and $\tan \beta = 3.5$ (*left*), $\tan \beta = 10$ (*right*), shown in the *upper panels* of Fig. 6

**Fig. 8:**
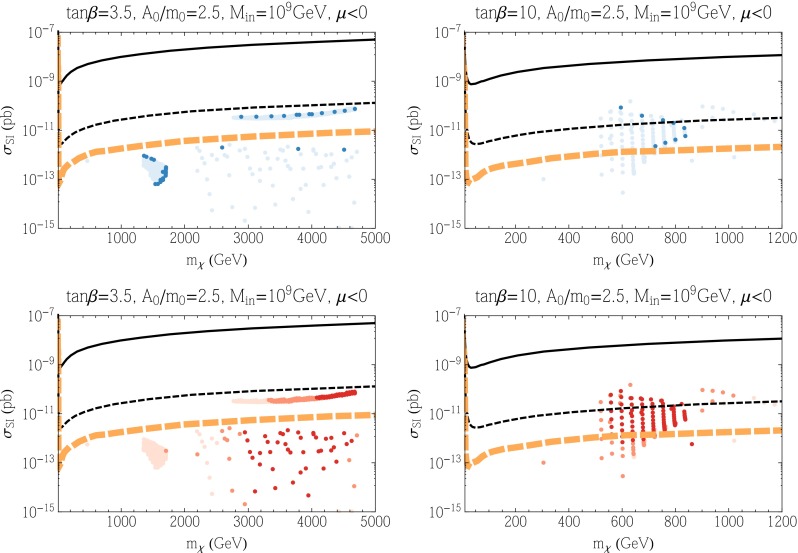
As in Fig. 3 for the subGUT case with $A_0 = 2.5 m_0$, $\mu < 0$, $M_{\mathrm{in}} = 10^9$ GeV and $\tan \beta = 3.5$ (*left*), $\tan \beta = 10$ (*right*), shown in the *lower panels* of Fig. 6

Results for the SI cross section for the subGUT CMSSM cases displayed in Fig. 6 are shown in Figs. 7 (for $\mu > 0$) and 8 (for $\mu < 0$). We see in the upper panels of Fig. 7 that the SI cross sections for models with a relic density compatible with the Planck range (darker blue points) are generally below the current upper limit but within reach of the LZ experiment. The dark-shaded points originate from what appears to be the stau coannihilation strip. As noted above, obtaining the correct relic density at such large LSP masses requires additional coannihilation and mass degeneracies. In this case, the Higgsinos also have masses comparable to the bino mass. Along this horizontal strip of points in the left panel of Fig. 7, the mass difference ($\mu - M_1$) is relatively constant and hence from Eq. (14), when $\tan \beta$ is small, we obtain a cross section which is relatively constant as well. The same is true for most of the underdense (paler blue) points for $\tan \beta = 10$ (upper right panel), but underdense points for $\tan \beta = 3.5$ may have SI cross sections below the LZ sensitivity though above the neutrino background level. The lower panels of Fig. 7 show that many of the points within reach of the LZ experiment have values of $m_h$ close to the experimental value. Figure 8 (for $\mu < 0$) exhibits somewhat lower values of the SI cross section, in general. Note that the cluster of points with $m_\chi \sim 1500$ GeV correspond to bino LSPs in the stop coannihilation region. These points do not appear when $\mu > 0$ as that region has $m_h < 122$ GeV and hence not included in our scan for elastic cross sections. Consequently, most models with a relic density compatible with the Planck range are beyond the LZ sensitivity, and some of the $\tan \beta = 3.5$ points are below the neutrino background level. The same is true a fortiori for the points with underdense relic neutralinos. Finally we note in passing, that the three nearly horizontal points below the neutrino background (for $\mu < 0$) originate from the funnel region (there are similar points when $\mu > 0$ but more difficult to discern in the figure).

**Fig. 9:**
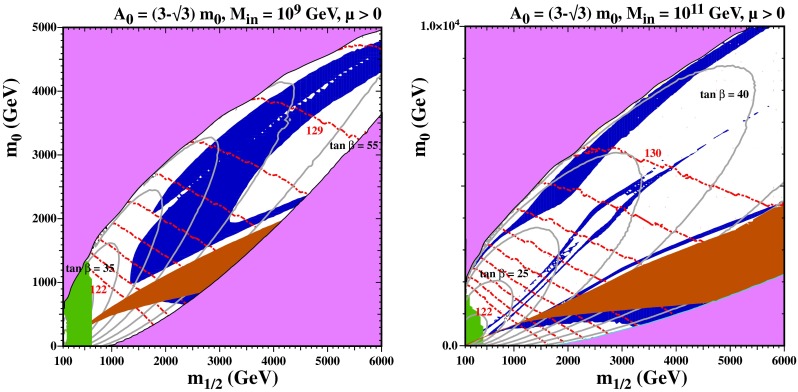
As in Fig. 2 for the subGUT mSUGRA case with $A_0 = (3-\sqrt{3}) m_0$, and $M_{\mathrm{in}} = 10^9$ GeV (*left*) and $M_{\mathrm{in}} = 10^{11}$ GeV (*right*). In addition to the shadings described for Fig. 2, the *green shaded region* is excluded by $b \rightarrow s \gamma$, the *gray lines* show *contours* of $\tan \beta$ in increments of 5 as labeled

Turning now to subGUT mSUGRA models, we consider only the Polonyi model, i.e., $A_0=(3-\sqrt{3})m_0$, with $\mu > 0$. The left panel of Fig. 9 is for $M_{\mathrm{in}}=10^9$ GeV, with contours of $\tan \beta$ in gray. The mauve shaded regions in the upper left and lower right parts of the plane are where electroweak symmetry breaking fails ($\mu ^2 < 0$ in the upper left and a diverging Yukawa coupling due to an excessive value for $\tan \beta$ in the lower right), and in the central brown shaded region the stau is the LSP. Above this region, various processes contribute to bringing the relic density into the Planck range. Over much of this plane, the LSP is mostly Higgsino: this is nearly degenerate with the next-to-lightest superparticle (NLSP), which is a chargino in this case, as well as the second Higgsino. In the blue shaded area above the stau LSP region, in addition to neutralino coannihilations, stau coannihilation also enhances the cross section in this strip. In the wide blue shaded region above the stau strip (recall that we are here showing regions where the relic density lies between 0.06 and 0.2) the Higgs funnel (lower part of this strip and conventional focus-point region (upper part) have merged. Below the stau LSP region, the gravitino becomes the LSP and can be dark matter. The values of $\tan \beta$ are quite large for all points in the left panel of Fig. 9. For this reason, the proton lifetime in minimal SU(5) models is much too short, and some non-minimal model must be considered.^5^ The right panel of Fig. 9 is for $M_{\mathrm{in}}=10^{11}$ GeV, and shares the qualitative features of the electroweak symmetry breaking and stau LSP constraints. The values of $\tan \beta$ are somewhat smaller than in the $M_{\mathrm{in}}=10^9$ GeV case, but still much too large to obtain a sufficiently long proton lifetime in minimal SU(5) models. The dark matter constraint is satisfied in a focus-point strip close to the electroweak symmetry-breaking boundary, which has now demerged from the funnel and stau coannihilation strip. The rapid-annihilation funnel is now clearly visible as a separate well-defined region. In the stau strip and in the funnel, the LSP is once again a bino, though the masses of the Higgsinos are not much larger. There is also a gravitino dark matter region below the stau LSP region.

**Fig. 10:**
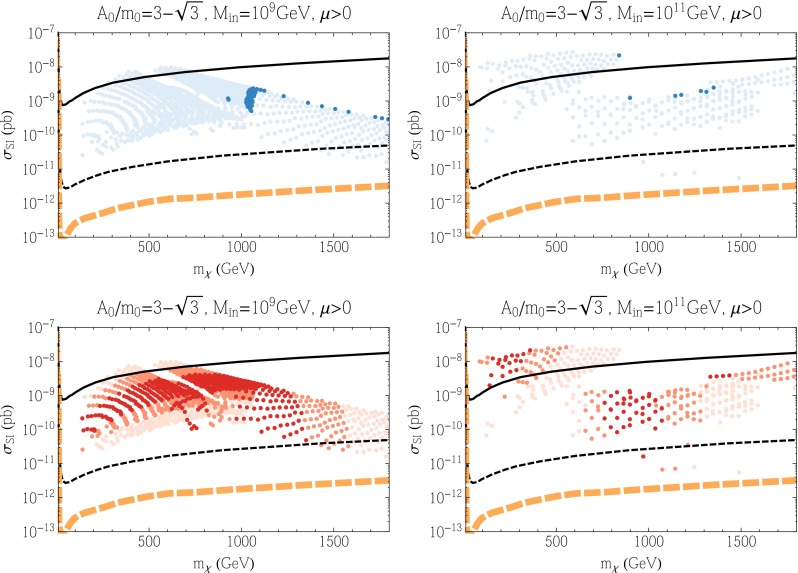
As in Fig. 3 for the subGUT mSUGRA case with $A_0 = (3 - \sqrt{3}) m_0$, and $M_{\mathrm{in}} = 10^9$ GeV (*left panels*) and $M_{\mathrm{in}} = 10^{11}$ GeV (*right panels*) shown in Fig. 9

Figure 10 displays results for the SI cross section in these subGUT mSUGRA models. We see in the upper panels that the SI cross section is generally between the current LUX upper limit and the prospective LZ sensitivity, though some models (particularly for $M_{\mathrm{in}}=10^{11}$ GeV) have cross sections above the LUX limit and a few underdense models have SI cross sections below the LZ sensitivity. For $M_{\mathrm{in}} = 10^9$ GeV, we clearly see the pile of points with the Planck relic density at $m_\chi \approx 1100$ GeV corresponding to a Higgsino LSP near the broad intersection of the stau strip and focus-point swath. The dark blue points in this figure continue to higher Higgsino masses along the stau coannihilation strip. Very low mass points (all lightly shaded) correspond to regions in the left panel of Fig. 9 that are to the left of the blue shaded region. In the white region to the left, the relic density is small, and in the white region to the right (between the stau strip and funnel) the relic density is too high. For $M_{\mathrm{in}} = 10^{11}$ GeV, we see two very distinct regions in Fig. 10. The region with lower masses ($m_\chi \lesssim 800$ GeV and cross section between $10^{-9}$ and $10^{-8}$ pb) originate from the focus-point region. The remainder of the points come from either the funnel or the stau strip and can be more easily distinguished by the lower panels showing the Higgs mass ranges. The relative paucity of dark-shaded blue points stems from the fact that the true Planck strips are quite thin in this case. Note also that there is no pile-up of points at 1100 GeV as the LSP is most bino rather than Higgsino at the higher value of $M_{\mathrm{in}}$. We see in the lower panels of this figure that models with $m_h \in [124, 126]$ GeV lie in the intersection region for $M_{\mathrm{in}} = 10^9$ GeV, and as noted above the dark brown shaded points for $M_{\mathrm{in}} = 10^{11}$ GeV at low masses come from the focus point whereas we now see that the middle group around $m_\chi \sim 1000$ GeV originate in the funnel, and the group at larger masses lie in the stau strip. All of the dark-shaded points lie within reach of the LZ experiment (though some $M_{\mathrm{in}}=10^{11}$ GeV models are excluded already by the LUX upper limit).

**Fig. 11:**
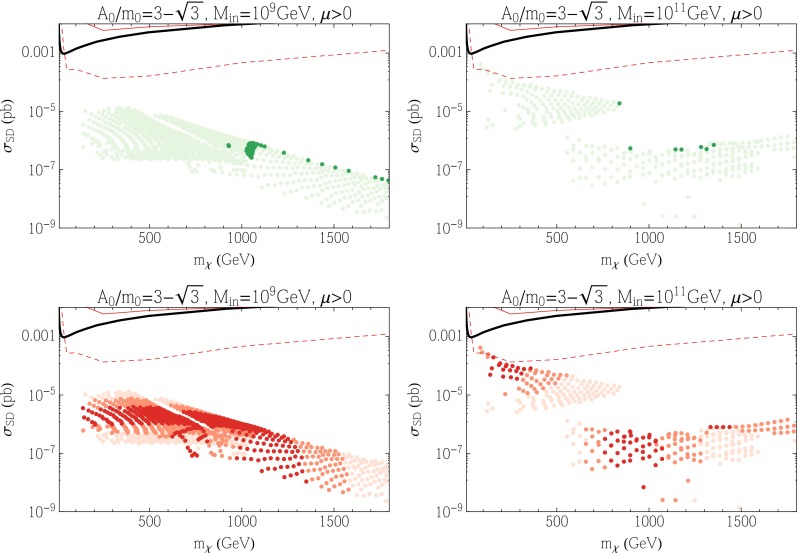
As in Fig. 3 for the subGUT mSUGRA case with $A_0 = (3 - \sqrt{3}) m_0$, and $M_{\mathrm{in}} = 10^9$ GeV (*left panels*) and $M_{\mathrm{in}} = 10^{11}$ GeV (*right panels*) shown in Fig. 9

We conclude this subsection by showing results for the SD cross sections in these subGUT mSUGRA models in Fig. 11. We see in the upper panels that the SD cross sections are generally smaller than the PICO bound [238], and also below the IceCube upper limits [239] for both ${\bar{b}} b$ and $W^+ W^-$ final states (which are likely to be more similar to the model final states). There is a handful of $M_{\mathrm{in}} = 10^{11}$ GeV models (most of them underdense) whose predictions lie close to the IceCube $W^+ W^-$ limit, but most model predictions are significantly below it. We see in the lower right panel of Fig. 11 that many of the models close to the IceCube $W^+ W^-$ limit have FeynHiggs$m_h$ values close to the experimental value. Comparing this figure with Fig. 10, it seems that there are better prospects for discovering SI scattering in these subGUT mSUGRA scenarios. However, we recall that these models yield proton lifetimes that are too short in minimal SU(5), pointing to the need for some non-minimal model.

### NUHM1

As mentioned earlier, in the NUHM1 one has the freedom to treat $\mu$ as a free parameter, and in the following we show $(m_{1/2}, m_0)$ planes for some representative choices of $\mu$, $\tan \beta$ and $A_0$. In fact, as long as $\tan \beta$ is small enough to obtain an acceptable proton lifetime, the qualitative behavior of the parameter space is relatively insensitive to $A_0$, though there is some dependence of the Higgs mass contours on $A_0$, as could be expected.

**Fig. 12:**
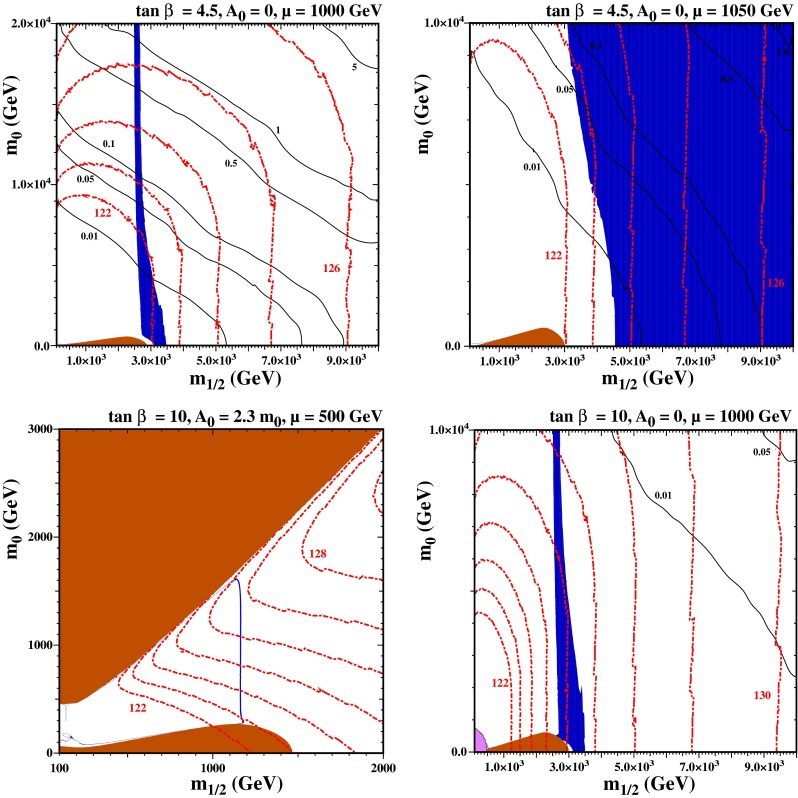
The NUHM1 $(m_{1/2}, m_0)$ planes for $\tan \beta = 4.5$ (*upper*) and $\tan \beta = 10$ (*lower*). We take $\mu = 1000$ GeV in the *upper left panel* and 1050 GeV in the *upper right panel*, both with $A_0 = 0$. In the *lower panels*, $\mu = 500$ GeV with $A_0 = 2.3 m_0$ (*left*) and $\mu = 1000$ GeV with $A_0 = 0$ (*right*). The *shading and contour* types are as in Fig. 2

We show in the upper left panel of Fig. 12 the $(m_{1/2}, m_0)$ plane for $\tan \beta = 4.5$, $A_0 = 0$ and $\mu = 1000$ GeV, which exhibits a small stau LSP region at low $m_0$ and $m_{1/2}$. Since $\mu$ is fixed, the composition of the LSP changes as $m_{1/2}$ is increased. At small $m_{1/2}$ the LSP is mainly bino and the relic density is too high. As $m_{1/2}$ is increased, the Higgsino component increases and the relic density passes through the Planck range across a relatively narrow, near-vertical transition strip. (Note that, in all four panels of this figure, the blue region corresponds to just the 3$\sigma$ Planck range rather than the extended range used in previous figures.) At larger $m_{1/2}$ the LSP is a Higgsino with a mass of about 1050 GeV which is slightly low for a Higgsino LSP and, as a result, the relic density is somewhat too small when $m_{1/2} \gtrsim 3$ TeV. In this panel, we see that we obtain an acceptable Higgs mass ($m_h > 124$ GeV) when $m_0 \gtrsim 13$ TeV. The proton lifetime is sufficiently large ($\tau _p \gtrsim 0.25 \times 10^{35}$ years) for this value of $m_0$.

In the upper right panel of Fig. 12, we have increased $\mu$ slightly to 1050 GeV. The most striking feature is that the dark matter region fills the right part of the plane: indeed, it extends infinitely far to the right toward large gaugino masses. In this case, when the gaugino mass is large, the LSP is a nearly pure Higgsino, as is the NLSP. This near-degeneracy facilitates coannihilation that brings the relic density within the acceptable range, with $\Omega _\chi h^2$ being determined predominantly by $\mu$ [136, 137]. The Higgsino mass in this case is very close to 1100 GeV, which remains constant at large $m_{1/2}$. Thus there is a very large (infinite) area where the relic density matches the Planck result. At low $m_{1/2}$, the relic density is too large and drops monotonically as the gaugino mass is increased and asymptotes to the Planck density at very large $m_{1/2}$. For $m_0 \lesssim 10$ TeV, when $A_0 = 0$, the Higgs mass contours are nearly vertical and the value of $\tan \beta = 4.5$ was chosen to maximize the area with good relic density and Higgs masses. The area between $m_{1/2} = 5$ TeV and 9 TeV has $m_h$ between 124 and 126 GeV, and increasing (decreasing) $\tan \beta$ by 0.5 would raise (lower) $m_h$ by roughly 1 GeV. Much of this region has $\tau _p \gtrsim 0.05 \times 10^{35}$ years: requiring $\tau _p > 5 \times 10^{33}$ years implies either $m_{1/2} \gtrsim 7.8$ TeV for small $m_0$ or $m_0 \gtrsim 8$ TeV for $m_{1/2} \simeq 4$ TeV. As $\mu$ is increased past 1050 GeV, the left edge of the blue shaded region moves quickly to the right and the relic density would be too large over much of the plane. The relic density would now asymptote to a value in excess of the Planck density. The Higgs mass is independent of $A_0/m_0$ for small $m_0$ but the Higgs mass contours bend to the left as $A_0/m_0$ is increased, so that the Higgs mass becomes large at larger $m_0$.

In the lower panels of Fig. 12, we have taken $\tan \beta = 10$. In the left panel, $\mu = 500$ and the transition strip from bino to Higgsino dark matter is much narrower and occurs at much lower $m_{1/2} \approx 1200$ GeV. Had we chosen $A_0 = 0$ as in the previous plots, the Higgs mass would be far too small. This can be compensated in this panel by choosing larger $A_0$, and we have chosen $A_0 = 2.3 m_0$ in this panel. As in the CMSSM, there is now a shaded region where the LSP is a stop in the upper left of the panel. There is a barely visible stop coannihilation strip that runs close to the stop LSP boundary, from the transition strip down to smaller $m_{1/2}$ and $m_0$. There is also a narrow stau coannihilation strip running on top of the stau LSP region at low $m_0$. In the right panel, we have again taken $A_0 = 0$ and increased $\mu$ to 1000 GeV. The relic density region resembles that in the upper left panel of the same figure, though the Higgs masses are now notably larger. The transition strip is now centered on $m_h = 126$ GeV, which is compatible within the experimental measurement within the theoretical uncertainties. We note also that the proton lifetime is far smaller in the lower panels due to the larger value of $\tan \beta$. Indeed, in the lower left panel $\tau _p$ is always below $0.01 \times 10^{35}$ years.

**Fig. 13:**
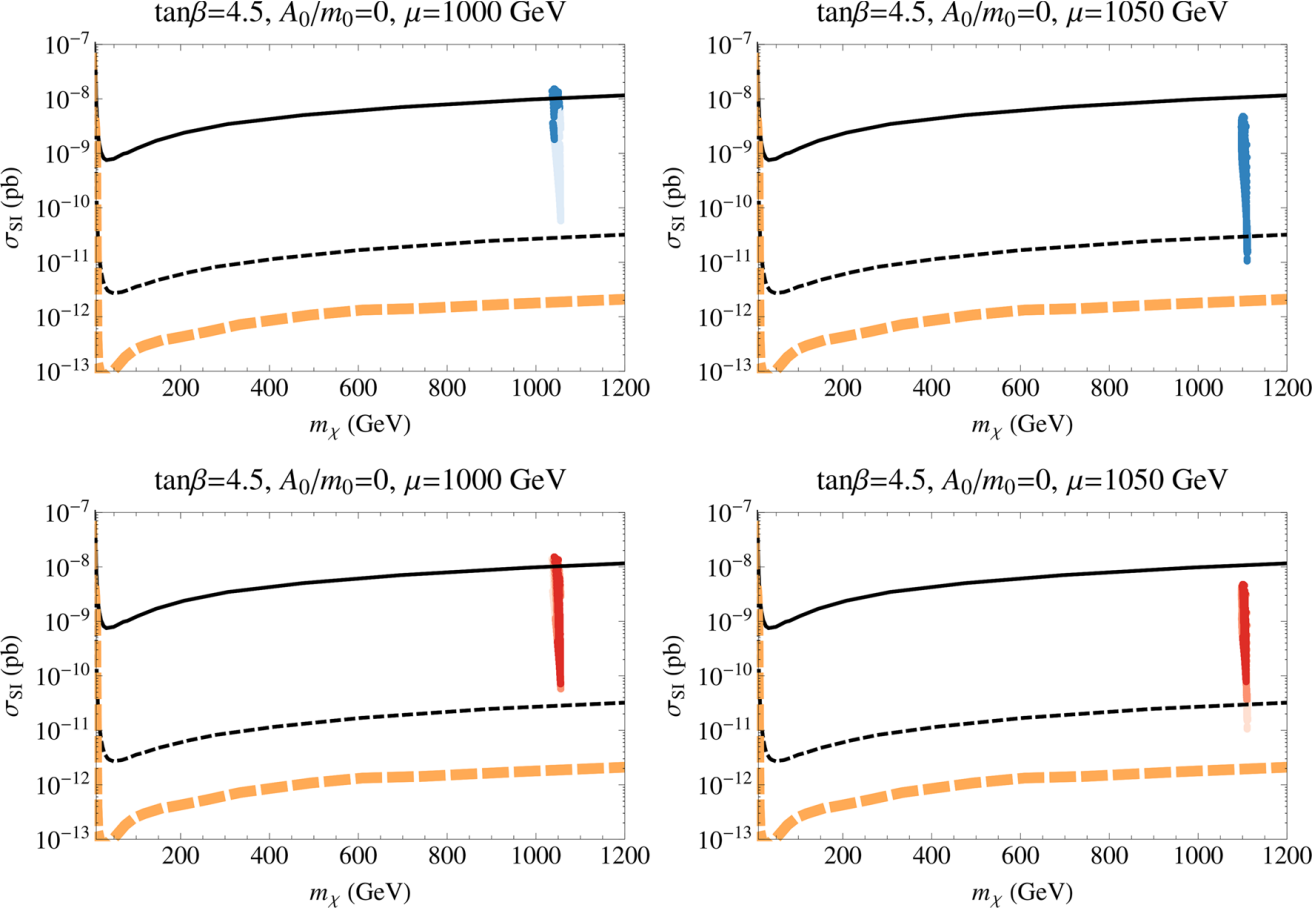
As in Fig. 3 for the NUHM1 cases with $\tan \beta = 4.5$ and $A_0 = 0$ with $\mu = 1000$ GeV (*left*) and $\mu = 1050$ GeV (*right*)

**Fig. 14:**
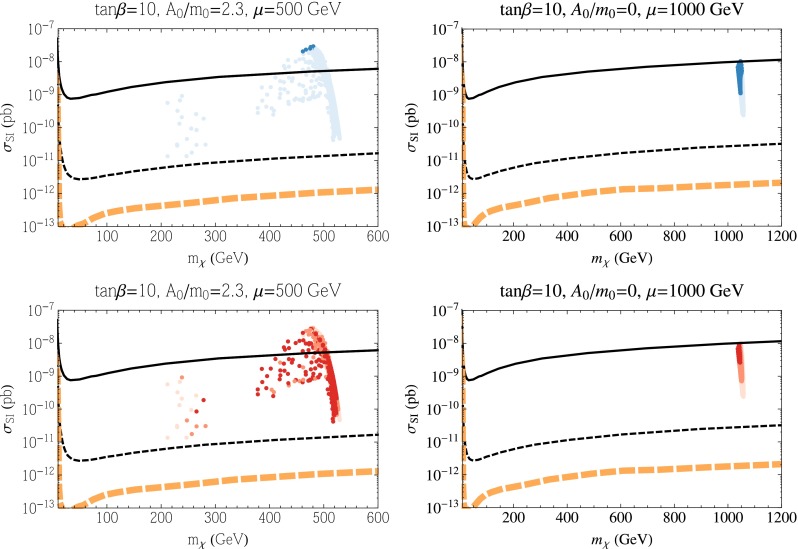
As in Fig. 3 for the NUHM1 with $\tan \beta = 10$ and $A_0 = 2.3 m_0$ with $\mu = 500$ GeV (*left*) and $A_0 = 0$ with $\mu =1000$ GeV (*right*)

The elastic scattering cross sections for the four panels of Fig. 12 are shown in Figs. 13 and 14. The left panels of Fig. 13 correspond to the NUHM1 model with $\tan \beta = 4.5$, $A_0 = 0$, and $\mu = 1000$ GeV. Viable points (with the correct relic density or less) have gaugino masses of around 3 TeV (for the correct relic density) or greater (less than the Planck density). In either case, the LSP mass is just over 1 TeV, which explains why all the points line up vertically. Most of the points (though not all) lie below the current LUX limit and all of them lie above the LZ projected reach. Note that, in principle, this vertical strip could extend further down, into the neutrino background, if we continued to sample points at higher $m_{1/2}$. Our sampling of points includes only points with $m_h$ between 122 and 128 GeV. Concerning the right panels with $\mu = 1050$ GeV, we recall that much of the $(m_{1/2}, m_0)$ plane contains a Higgsino LSP with the desired relic density. In that region, the mass of the LSP is always very close to 1100 GeV and that fact is readily seen in the right panels of Fig. 13, where all the points stack vertically at $m_\chi \simeq 1100$ GeV. All of these points lie below the current LUX bound, but most of them are within the projected reach of LZ. As in the previous example, the vertical strip of points could go lower if we sampled to larger $m_{1/2}$ where $m_h > 128$ GeV. As seen in the lower right panel of this figure, the points with $m_h$ between 124 and 126 GeV are all accessible to LZ.

**Fig. 15:**
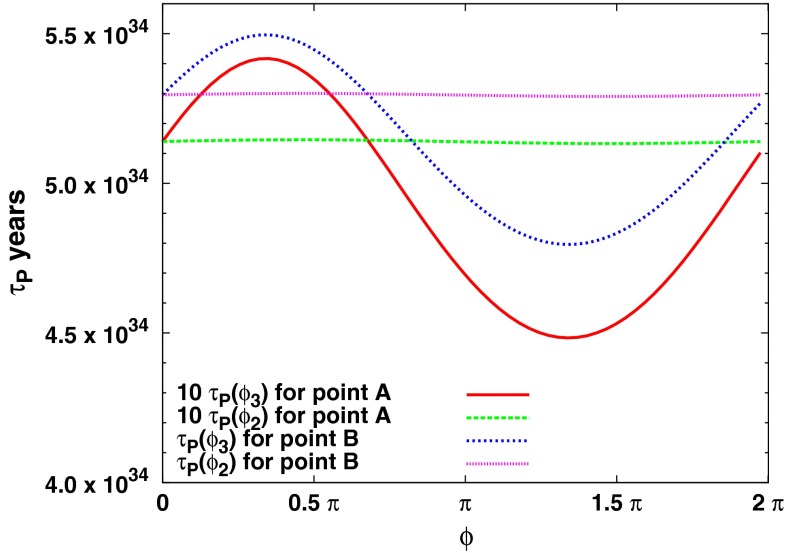
The variation of the proton lifetime due to the phases of the Wilson coefficients for point A ($m_{1/2}= 2.2$ TeV, $m_0=10$ TeV, $A_0=0$, $\tan \beta =5$ and $M_{\mathrm{in}}=M_{\mathrm{GUT}}$) and point B ($m_{1/2}=8$ TeV, $m_0=5.2$ TeV, $A_0=2.5m_0$, $\tan \beta =3.5$, and $M_{\mathrm{in}}=10^9$ GeV)

In Fig. 15, we show the spin-independent cross sections for $\tan \beta = 10$. In the left panels with $\mu = 500$ GeV, we again see a pile-up at a fixed LSP mass, $m_\chi \sim 500$ GeV, corresponding in this case to the transition strip and the region to its right. Since the strip is so narrow in this case, there are very few dark-shaded points, and these have cross sections that exceed the LUX bound. There are also a few points at lower $m_\chi$ that originate near the stop or stau coannihilation strips. All of the points shown lie within the LZ projected reach. One also sees in the lower left panel that many of the points have a Higgs mass in the 124–126 GeV range. Indeed, for an underdense Higgsino-like LSP with $m_\chi \approx 500$ GeV (to the right of the transition strip in Fig. 12), there are also many points with $m_h>126$ GeV, though these points are eclipsed in Fig. 15 by those with more favorable $m_h$. In the right panels of Fig. 15, for $\mu = 1000$ GeV, all points in the transition strip and to the right of the strip have a narrow range of LSP masses fixed by the value of $\mu$. These points lie just below the current LUX bound. Points that are most compatible with Planck dark matter abundance and have $m_h \approx 125$ GeV have the largest SI cross sections, within an order of magnitude of the current LUX limit. All points considered here, with $122 <m_h<128$ GeV, should be accessible with LZ.

## Discussion

Large parts of the CMSSM parameter space are excluded by the absence (so far) of proton decay, if the CMSSM is embedded within the minimal SU(5) GUT. There are regions of parameter space with $\tan \beta \lesssim 5$ and $(m_{1/2}, m_0) \gtrsim$ several TeV that are still allowed, however. Typically, these models predict a spin-independent dark matter-nucleon scattering cross section that falls below the current LUX upper limit but could be accessible to the planned LZ experiment. In fact, it is the constraints coming from the Higgs boson mass which exclude the bulk of the model points which are beyond the reach of LZ. The prospects for direct detection of spin-independent dark matter scattering are reduced for $\mu < 0$ and for $A_0 > 0$, and the cross sections for spin-dependent dark matter scattering are generally substantially below the current bounds from PICO and IceCube.

In view of the limited perspectives within the CMSSM, we have explored in this paper the prospects for probing other MSSM scenarios via proton decay and dark matter detection. In one class of scenarios, called subGUT models, universality of the soft supersymmetry-breaking masses is retained, but it is imposed at some scale $M_{\mathrm{in}} < M_{\mathrm{GUT}}$. Within the subGUT CMSSM there are more possibilities for bringing the supersymmetric relic density within the range allowed by Planck and other experiments even if $m_{1/2}$ and $m_0$ are each several TeV, thanks in particular to the more compressed spectrum and consequently the greater possibilities for coannihilation processes that bring the dark matter density down into the allowed range. However, small values of $\tan \beta \lesssim 5$ are preferred again, as in the GUT-scale CMSSM, unless one adopts a non-minimal GUT structure. For $M_{\mathrm{in}} = 10^9$ GeV and $\tan \beta = 3.5$ or 10, we find spin-independent dark matter-nucleon scattering cross sections that are well within the range allowed by LUX, and the spin-independent cross section may fall below the neutrino background level, particularly for $\mu < 0$.

In mSUGRA models, the possibilities are very limited if $M_{\mathrm{in}} = M_{\mathrm{GUT}}$ but open up for $M_{\mathrm{in}} < M_{\mathrm{GUT}}$. On the other hand, in mSUGRA models $\tan \beta$ is no longer a free parameter, and the electroweak vacuum conditions typically require large values that give severe problems within the minimal SU(5) GUT framework. That said, spin-independent dark matter scattering may again lie within reach of the LZ experiment, whereas spin-dependent scattering cross sections generally lie below the PICO and IceCube upper limits.

In the NUHM1 one may regard the Higgs mixing parameter $\mu$ as an extra free parameter compared to the CMSSM. This freedom opens up new possibilities for models that respect the dark matter, Higgs mass, and proton decay constraints. In particular, since varying $\mu$ varies the Higgsino component of the LSP, there is the possibility of a ‘well-tempered’ transition region as well as the more familiar stau and stop coannihilation possibilities for bringing the relic neutralino density into (or below) the Planck range. Moreover, for some $\mu$ values as seen in the upper right panel of Fig. 12, in particular, the relic density may lie within the Planck range up to indefinitely high values of $m_{1/2}$ and $m_0$. In this case, the proton lifetime may certainly be long enough to survive the present experimental lower limit whereas, as seen in Fig. 13, the spin-independent dark matter scattering cross section is likely to be within reach of the planned LZ experiment.

In conclusion, the examples studied in this paper show that there are certainly interesting possibilities for probing supersymmetric models beyond the CMSSM via searches for proton decay and direct dark matter scattering.

## Appendix A: Wilson coefficients and RGEs for proton decay

In this appendix, we summarize the matching conditions and RGEs used in the proton decay calculation discussed in Sect. 3.1. The Wilson coefficients $C^{ijkl}_{5L}$ and $C^{ijkl}_{5R}$ in Eq. (6) are given at the GUT scale by

21 $$\begin{aligned} C^{ijkl}_{5L}(M_{\mathrm{GUT}})&=\frac{1}{M_{H_C}}y_{u_i} \mathrm{e}^{i\varphi _i}\delta ^{ij}V^*_{kl}y_{d_l},\nonumber \\ C^{ijkl}_{5R}(M_{\mathrm{GUT}})&=\frac{1}{M_{H_C}}y_{u_i}V_{ij}V^*_{kl}y_{d_l} \mathrm{e}^{-i\varphi _k}, \end{aligned}$$

where $$y_{u_i}$$ and $$y_{d_l}$$ are up- and down-type Yukawa couplings at the GUT scale, respectively, $$V_{ij}$$ is the Cabibbo–Kobayashi–Maskawa (CKM) matrix, and $$\varphi _i$$$$(i=1,2,3)$$ denote the extra phases appearing in the GUT Yukawa couplings. They are taken such that they satisfy $$\sum _{i}\varphi _i = 0$$, and thus there are two independent degrees of freedom [195]. For the definition of the GUT Yukawa couplings, we follow the convention of [116]. These unknown phases cause uncertainty in our calculation, whose significance is estimated below.

These Wilson coefficients are run down to the SUSY-breaking scale using RGEs. Since the theory is supersymmetric, the RGEs of the Wilson coefficients are readily obtained from the anomalous dimensions of the fields in the corresponding effective operators, thanks to the non-renormalization property [240] of the holomorphic operators. Hence we have

22 $$\begin{aligned} \frac{\mathrm{d}}{\mathrm{d}\ln Q} C^{ijkl}_{5L}&=\frac{1}{16\pi ^2}\times \biggl [-\frac{2}{5}g_1^2 -6g_2^2 -8g_3^2+y_{u_i}^2 +y_{d_i}^2\nonumber \\&\quad + y_{u_j}^2 +y_{d_j}^2 + y_{u_k}^2 + y_{d_k}^2 + y_{e_l}^2 \biggr ]C^{ijkl}_{5L}, \nonumber \\ \frac{\mathrm{d}}{\mathrm{d}\ln Q} C^{ijkl}_{5R}&=\frac{1}{16\pi ^2}\biggl [-\frac{12}{5}g_1^2 -8g_3^2 +2y_{u_i}^2 + 2y_{e_j}^2 \nonumber \\&\quad + 2y_{u_k}^2 + 2y_{d_l}^2 \biggr ] C^{ijkl}_{5R}, \end{aligned}$$

where *Q* denotes the renormalization scale.

The matching conditions at the sfermion mass scale are

23 $$\begin{aligned} C_i^{\widetilde{H}}&=\frac{y_ty_\tau }{(4\pi )^2} F(\mu , m_{\widetilde{t}_R}^2,m_{\tau _R}^2) C^{*331i}_{5R}, \nonumber \\ C^{\widetilde{W}}_{jk}&= \frac{\alpha _2}{4\pi }\left[ F(M_2, m_{\widetilde{Q}_1}^2, m_{\widetilde{Q}_j}^2) +F(M_2, m_{\widetilde{Q}_j}^2, m_{\widetilde{L}_k}^2)\right] C^{jj1k}_{5L}, \nonumber \\ \overline{C}^{\widetilde{W}}_{jk}&= -\frac{3}{2} \frac{\alpha _2}{4\pi }\left[ F(M_2, m_{\widetilde{Q}_j}^2, m_{\widetilde{Q}_j}^2) +F(M_2, m_{\widetilde{Q}_1}^2, m_{\widetilde{L}_k}^2)\right] C^{jj1k}_{5L}, \end{aligned}$$

where $m_{\widetilde{t}_R}$, $m_{\widetilde{\tau }_R}$, $m_{\widetilde{Q}_j}$, and $m_{\widetilde{L}_k}$ are the masses of the right-handed stop, the right-handed stau, left-handed squarks, and left-handed sleptons, respectively, $\alpha _i \equiv g_i^2/(4\pi )$, and

24 $$\begin{aligned} F(M, m_1^2, m_2^2)&\equiv \frac{M}{m_1^2-m_2^2} \biggl [\frac{m_1^2}{m_1^2-M^2}\ln \biggl (\frac{m_1^2}{M^2}\biggr ) \nonumber \\&\quad -\frac{m_2^2}{m_2^2-M^2}\ln \biggl (\frac{m_2^2}{M^2}\biggr ) \biggr ]. \end{aligned}$$

Between the SUSY-breaking scale and the electroweak scale, the RGEs for the Wilson coefficients are given by [199]

25 $$\begin{aligned} \frac{\mathrm{d}}{\mathrm{d}\ln Q} C^{\widetilde{H}}_{i}&= \biggl [\frac{\alpha _1}{4\pi }\biggl (-\frac{11}{10}\biggr ) +\frac{\alpha _2}{4\pi }\biggl (-\frac{9}{2}\biggr )\nonumber \\&\quad +\frac{\alpha _3}{4\pi }(-4) +\frac{1}{2}\frac{f_{t}^2}{16\pi ^2} \biggr ]C^{\widetilde{H}}_{i},\nonumber \\ \frac{\mathrm{d}}{\mathrm{d}\ln Q} C^{\widetilde{W}}_{jk}&= \biggl [\frac{\alpha _1}{4\pi }\biggl (-\frac{1}{5}\biggr ) +\frac{\alpha _2}{4\pi }(-3)\nonumber \\&\quad +\frac{\alpha _3}{4\pi }(-4) +\frac{f_{u_j}^2}{16\pi ^2} \biggr ]\nonumber \\&\quad \times C^{\widetilde{W}}_{jk} +\frac{\alpha _2}{4\pi }(-4)[ 2C^{\widetilde{W}}_{jk} + \overline{C}^{\widetilde{W}}_{jk}],\nonumber \\ \frac{\mathrm{d}}{\mathrm{d}\ln Q} \overline{C}^{\widetilde{W}}_{jk}&= \biggl [\frac{\alpha _1}{4\pi }\biggl (-\frac{1}{5}\biggr ) +\frac{\alpha _2}{4\pi }(-3) \nonumber \\&\quad +\frac{\alpha _3}{4\pi }(-4) +\frac{f_{u_j}^2}{16\pi ^2} \biggr ]\nonumber \\&\quad \times \overline{C}^{\widetilde{W}}_{jk} +\frac{\alpha _2}{4\pi }(-4)[ 2C^{\widetilde{W}}_{jk} + \overline{C}^{\widetilde{W}}_{jk}], \end{aligned}$$

where the $f_{u_j}$ denote the SM up-type Yukawa couplings. At the electroweak scale, the effective operators are matched onto the effective interactions that induce the $p\rightarrow K^+\bar{\nu }_k$ decay mode. The interactions are written as

26 $$\begin{aligned} \mathcal{L}(p\rightarrow K^+\bar{\nu }_i^{})&=C_{RL}(usd\nu _i)\bigl [\epsilon _{abc}(u_R^as_R^b)(d_L^c\nu _i^{})\bigr ]\nonumber \\&\quad +C_{RL}(uds\nu _i)\bigl [\epsilon _{abc}(u_R^ad_R^b)(s_L^c\nu _i^{})\bigr ] \nonumber \\&\quad +C_{LL}(usd\nu _i)\bigl [\epsilon _{abc}(u_L^as_L^b)(d_L^c\nu _i^{})\bigr ]\nonumber \\&\quad +C_{LL}(uds\nu _i)\bigl [\epsilon _{abc}(u_L^ad_L^b)(s_L^c\nu _i^{})\bigr ], \end{aligned}$$

and we have

27 $$\begin{aligned} C_{RL}(usd\nu _\tau )&=-V_{td}C^{\widetilde{H}}_{2}(m_Z),\nonumber \\ C_{RL}(uds\nu _\tau )&=-V_{ts}C^{\widetilde{H}}_{1}(m_Z),\nonumber \\ C_{LL}(usd\nu _k)&=\sum _{j=2,3}V_{j1}V_{j2} C^{\widetilde{W}}_{jk}(m_Z),\nonumber \\ C_{LL}(uds\nu _k)&=\sum _{j=2,3}V_{j1}V_{j2} C^{\widetilde{W}}_{jk}(m_Z). \end{aligned}$$

We note that $\overline{C}^{\widetilde{W}}_{jk}$ does not contribute to the electroweak matching conditions: it is relevant only to the RGEs.

The above coefficients are then run down to the hadronic scale $Q_{\text {had}}= 2$ GeV, where the matrix elements of the effective operators are evaluated. The QCD contributions to the RGE for this step are calculated at two-loop level in Ref. [200]. For a generic coefficient *C*, the two-loop RGE is

28 $$\begin{aligned} \frac{\mathrm{d}}{\mathrm{d} \ln Q}C = -\biggl [ 4\frac{\alpha _s}{4\pi }+\biggl (\frac{14}{3}+\frac{4}{9}N_f +\Delta \biggr )\frac{\alpha _s^2}{(4\pi )^2} \biggr ]C, \end{aligned}$$

where $\alpha _s$ is the strong coupling constant, $N_f$ is the number of quark flavors, and $\Delta =0$ ($\Delta =-10/3$) for $C_{LL}$ ($C_{RL}$). The analytical solutions of the RGEs are given in Refs. [115, 200].

For the hadron matrix elements of the effective operators, we use the results given by the lattice QCD simulation in [201]. Using these results, we obtain finally the partial decay width of the $p\rightarrow K^+ \bar{\nu }_i$ mode:

29 $$\begin{aligned} \Gamma (p\rightarrow K^+\bar{\nu }_i) =\frac{m_p}{32\pi }\biggl (1-\frac{m_K^2}{m_p^2}\biggr )^2 \vert \mathcal{A}(p\rightarrow K^+\bar{\nu }_i)\vert ^2,\nonumber \\ \end{aligned}$$

where $m_p$ and $m_K$ are the masses of the proton and kaon, respectively. The amplitude $\mathcal{A}(p\rightarrow K^+\bar{\nu }_i)$ is the sum of the Wilson coefficients multiplied by the corresponding hadron matrix elements:

30 $$\begin{aligned} \mathcal{A}(p\rightarrow K^+\bar{\nu }_e)&= C_{LL}(usd\nu _e)\langle K^+\vert (us)_Ld_L\vert p\rangle \nonumber \\&\quad +C_{LL}(uds\nu _e)\langle K^+\vert (ud)_Ls_L\vert p\rangle , \nonumber \\ \mathcal{A}(p\rightarrow K^+\bar{\nu }_\mu )&= C_{LL}(usd\nu _\mu )\langle K^+\vert (us)_Ld_L\vert p\rangle \nonumber \\&\quad +C_{LL}(uds\nu _\mu )\langle K^+\vert (ud)_Ls_L\vert p\rangle , \nonumber \\ \mathcal{A}(p\rightarrow K^+\bar{\nu }_\tau )&= C_{RL}(usd\nu _\tau )\langle K^+\vert (us)_Rd_L\vert p\rangle \nonumber \\&\quad + C_{RL}(uds\nu _\tau )\langle K^+\vert (ud)_Rs_L\vert p\rangle \nonumber \\&\quad + C_{LL}(usd\nu _\tau )\langle K^+\vert (us)_Ld_L\vert p\rangle \nonumber \\&\quad +C_{LL}(uds\nu _\tau )\langle K^+\vert (ud)_Ls_L\vert p\rangle . \end{aligned}$$

The following are the numerical values of the hadron matrix elements at the scale of $Q_{\text {had}}=2$ GeV found in [201]:

31 $$\begin{aligned} \langle K^+\vert (us)_L ^{}d_L^{}\vert p\rangle&= 0.036(12)(7) ~~\text {GeV}^2, \nonumber \\ \langle K^+\vert (ud)_L ^{}s_L^{}\vert p\rangle&= 0.111(22)(16) ~~\text {GeV}^2, \nonumber \\ \langle K^+\vert (us)_R ^{}d_L^{}\vert p\rangle&= -0.054(11)(9) ~~\text {GeV}^2, \nonumber \\ \langle K^+\vert (ud)_R ^{}s_L^{}\vert p\rangle&= -0.093(24)(18) ~~\text {GeV}^2. \end{aligned}$$

The first and second parentheses represent statistical and systematic errors, respectively.

As mentioned above, new phases appearing in the GUT Yukawa couplings yield uncertainty in the calculation [185], though we have neglected the effects of these phases in the main text. To justify our neglect of these parameters, here we will show the variation in the proton lifetime when the phases of the Wilson coefficients are included. We select two points for analysis and present their phase dependence in Fig. 15. Point A is for $m_{1/2}= 2.2$ TeV, $m_0=10$ TeV, $A_0=0$, $\tan \beta =5$ and $M_{\mathrm{in}}=M_{\mathrm{GUT}}$ and has been labeled in Fig. 2. Point B is for $m_{1/2}=8$ TeV, $m_0=5.2$ TeV, $A_0=2.5m_0$, $\tan \beta =3.5$, and $M_{\mathrm{in}}=10^9$ GeV and has been labeled in Fig. 6. The variation arises due to cancellations between the wino and Higgsino contribution to the proton decay. Diagrams representing these processes are found in Fig. 1. As is clearly seen from Fig. 15, the variation is small in comparison to the uncertainty coming from the Yukawa couplings. Thus, we ignore these effects in the main text.

## Footnotes

1 Note that we do not impose any constraint from the anomalous magnetic moment of the muon, $g_\mu - 2$ [96, 97].

2 We use the same notation for the chiral superfields and their spin-zero components.

3 The effects on proton decay of possible flavor violation in the sfermion sector are discussed in [115].

4 This choice corresponds to $y\equiv 2\langle N|\overline{s}s|N\rangle /\langle N| \overline{u}u + \overline{d}d |N\rangle = 0.28$, which is larger than the values obtained in lattice QCD simulations [216–219]. We note that the uncertainties in these quantities (see, e.g., [220–222]) affect significantly the resultant scattering cross sections [212].

5 We note also that the $b \rightarrow s \gamma$ constraint excludes a region at small $m_{1/2}$ and $m_0$ (shaded green), and that the $B_s \rightarrow \mu ^+ \mu ^-$ constraint is also relevant in much of the allowed region of the plane.
